# Constructing a Theory- and Evidence-Based Treatment Rationale for Complex eHealth Interventions: Development of an Online Alcohol Intervention Using an Intervention Mapping Approach

**DOI:** 10.2196/resprot.2371

**Published:** 2013-01-23

**Authors:** Håvar Brendryen, Ayna Johansen, Sverre Nesvåg, Gerjo Kok, Fanny Duckert

**Affiliations:** ^1^The Norwegian Centre for Addiction Research, Institute of Clinical MedicineFaculty of MedicineUniversity of OsloOsloNorway; ^2^Alcohol and Drug Research Western NorwayStavanger University HospitalStavangerNorway; ^3^Department of Work and Social PsychologyMaastricht UniversityMaastrichtNetherlands

**Keywords:** early intervention, at-risk drinkers, hazardous drinking, harmful drinking, intervention mapping, Internet, cell phone, eHealth, short message service

## Abstract

**Background:**

Due to limited reporting of intervention rationale, little is known about what distinguishes a good intervention from a poor one. To support improved design, there is a need for comprehensive reports on novel and complex theory-based interventions. Specifically, the emerging trend of just-in-time tailoring of content in response to change in target behavior or emotional state is promising.

**Objective:**

The objective of this study was to give a systematic and comprehensive description of the treatment rationale of an online alcohol intervention called Balance.

**Methods:**

We used the intervention mapping protocol to describe the treatment rationale of Balance. The intervention targets at-risk drinking, and it is delivered by email, mobile phone text messaging, and tailored interactive webpages combining text, pictures, and prerecorded audio.

**Results:**

The rationale of the current treatment was derived from a self-regulation perspective, and the overarching idea was to support continued self-regulation throughout the behavior change process. Maintaining the change efforts over time and coping adaptively during critical moments (eg, immediately before and after a lapse) are key factors to successful behavior change. Important elements of the treatment rationale to achieving these elements were: (1) emotion regulation as an inoculation strategy against self-regulation failure, (2) avoiding lapses by adaptive coping, and (3) avoiding relapse by resuming the change efforts after a lapse. Two distinct and complementary delivery strategies were used, including a day-to-day tunnel approach in combination with just-in-time therapy. The tunnel strategy was in accordance with the need for continuous self-regulation and it functions as a platform from which just-in-time therapy was launched. Just-in-time therapy was used to support coping during critical moments, and started when the client reports either low self-efficacy or that they were drinking above target levels.

**Conclusions:**

The descriptions of the treatment rationale for Balance, the alcohol intervention reported herein, provides an intervention blueprint that will aid in interpreting the results from future program evaluations. It will ease comparisons of program rationales across interventions, and may assist intervention development. By putting just-in-time therapy within a complete theoretical and practical context, including the tunnel delivery strategy and the self-regulation perspective, we have contributed to an understanding of how multiple delivery strategies in eHealth interventions can be combined. Additionally, this is a call for action to improve the reporting practices within eHealth research. Possible ways to achieve such improvement include using a systematic and structured approach, and for intervention reports to be published after peer-review and separately from evaluation reports.

## Introduction

Improved reporting of intervention rationales within eHealth will extend the evidence base and may improve the design of future intervention programs [1-9]. Research and development teams that set out to create interventions are informed in 2 basic ways—directly, based on empirical reports, and indirectly, through systematic or meta-analytic reviews. However, descriptions of the treatment rationales within empirical reports, including theoretical backdrop, treatment goals, causal mechanisms, behavior change techniques, delivery, and content, tend to be confined to a few paragraphs in the methods section [1]. Researchers need to be able to compare the treatment rationale across programs to adequately interpret the results from the empirical findings of the field. Therefore, the confined descriptions disable researchers who need to form hypotheses about how to improve intervention design during the process of creating new interventions [10]. Systematic reports of interventions should inspire creators of eHealth interventions toward use of a broader array of methods, just as treatment manuals do for clinicians who practice face-to-face psychotherapy [11]. The lack of detail in empirical reports also limits the insights possible within systematic reviews [2,3,12]. The findings from such reviews thus tend to be broad and lack detail. For example, a high quality review reported that “more extensive use of theory was associated with increases in effect size interventions that incorporated more behavior change techniques also tended to have larger effect…and the effectiveness of Internet-based interventions was enhanced by the use of additional methods of communicating” [12]. The insufficiency of these insights within systematic reviews is not due to the reviewers, but due to the limited reporting. As such, incomplete reports prevent reviewers from examining detailed hypotheses concerning the relationship between the intervention rationales and the outcomes [13].

Generally, we can say that eHealth interventions [14-19], such as alcohol interventions [20-22], show promise in terms of feasibility and efficacy, but we know little about what distinguishes the effective from the less effective interventions [12,22-24]. To increase the opportunity to extract insight and knowledge from eHealth studies, and to ultimately advance intervention design, it is necessary to improve the reporting of intervention rationales. Hence, the aim of the current article is to give a systematic and comprehensive description of the treatment rationale of an alcohol intervention (Balance).

## Methods

This paper uses intervention mapping (IM) to give a systematic and comprehensive description of the treatment rationale. The IM protocol [1] provides a structured approach to develop and describe health programs. It is a logical, methodic, step-by-step procedure that helps researchers organize their thoughts as they move from theory and evidence to practice, and it provides tools to describe the development process. For each of the steps in the mapping process, theory and evidence is approached differently [25], meaning that different theories play different roles throughout the mapping process. The end product constitutes a comprehensive blueprint of the intervention and a detailed treatment rationale, that may facilitate reproducibility, support the interpretation of subsequent evaluation studies, and ease the comparison of treatment rationale across intervention [25,26].

The objective of the paper is to describe treatment rationale, hence, we focused on the 4 applicable steps of IM: (1) a brief needs assessment, (2) defining the goal and the objectives of the intervention, (3) identification of intervention methods and applications, and (4) developing the actual program materials. Step 2 is further broken down into 3 levels of detail, which are the overall program goal, the performance objectives, and the change objectives. The program goal is the result you want to achieve with the intervention (eg, to make at-risk drinkers drink less), the performance objectives describe the actual behaviors that each client must perform to reach the program goal, and the change objectives outlines what needs to change with regard to specific behavior determinants for the program participants to do each of the performance objectives. In this way, the performance objective is a specification of the program goal, and the change objective is a specification of the performance objectives. The end result of step 2 is a matrix in which the performance objectives are crossed with the behavior determinants to form a set of change objectives. This matrix constitutes the core of the treatment rationale of which the subsequent steps of the development process is based upon.

## Results

### Needs Assessment

Alcohol use is the third leading contributor to the global burden of disease [27]. Currently, there is a need for alcohol interventions [28-30]. Typically, only those with the most severe alcohol problems are treated, while those with moderate problems go undetected [31-33]. This is unfortunate because the majority of alcohol-related harm and socioeconomic cost is not attributable to drinkers with severe alcohol dependence, but to the much larger group of at-risk drinkers, a group that receives little treatment attention today [34,35]. Therefore, we decided to focus on the at-risk drinkers, which include both hazardous and harmful drinking [28]. Hazardous drinking refers to a pattern of use that is of public health significance despite of the absence of any current disorder, while harmful drinking refers to a pattern of use that is already causing health damage. There exists no good estimate of at-risk drinking prevalence among the general Norwegian population. However, in Sweden, a comparable nation with regard to alcohol consumption, the prevalence of at-risk drinking has been reported as 18% for men and 5% for women [36]. A study of the private sector workplaces in Norway revealed that 11% of both male and female workers met the criteria for hazardous drinking [37].

While face-to-face alcohol screening and brief interventions are effective in reducing alcohol consumption [38], diffusion into routine health care has been slow [28,39] and limited by general practitioners’ time, competency, and willingness to implement them [40,41]. Internet-based programs have several advantages to these procedures, including a potentially higher reach to lower cost, accessibility, 24/7 availability, convenience, and anonymity [42,43]. Due to the stigma of receiving alcohol treatment and the high number of at-risk drinkers, eHealth interventions seem particularly well suited to this population [32]. The most common design of online alcohol interventions is the single-session screening and feedback format [20-22]. Based on the indirect comparisons of systematic reviews, the efficacy of simple single session interventions tend to be outperformed by the more complex multi-session interventions [12,22]. Hence, there is a need for novel and complex approaches to such treatment. Specifically, the tailoring of intervention content with just-in-time therapy in response to self-reported change in target behavior or emotional state is a promising and emerging trend to eHealth design [2,44-54].

### Defining the Goals and Objectives of the Treatment Program

#### Defining the Overall Goals of the Program

With regard to the intended aims of an intervention, the first level is the program goal, and the result one wants to achieve with the program. The goal of the current program is to diminish health risk and mitigate negative consequences of alcohol by encouraging lowered alcohol consumption among at-risk drinkers (ie, hazardous and harmful drinkers). Stated differently, the intervention is intended to make at-risk drinkers drink less. The performance objectives, change objectives, and sub-aims of the intervention will specify how this goal can be achieved.

#### Defining the Performance Objectives of the Program

A specific intervention may target one or several performance objectives. Performance objectives make up the actual behaviors that each client must perform for the program goal to be reached and their behavior to be changed. A performance objective is a specification of the program goal, and it defines more precisely what it is to drink less, in terms of behavior. The goal of the program was to make at-risk drinkers drink less. Self-regulatory processes appear to play key roles in both the causes and effects of alcohol consumption [55], and the self-regulation perspective can account for several of the topics we believed to be important for this type of behavior change, like maintenance of the efforts to change over time, lapse and relapse prevention, as well as mood regulation. Hence, self-regulation theory was used to specify the program goal and identify the performance objectives. In this way, the overarching idea of the current treatment is to support continued self-regulation throughout the behavior change process. Although the self-regulation perspective was essential at this level of intervention development, other theories play important roles in subsequent steps of the development.

In a broad sense, self-regulation refers to any effort to alter personal responses, including thoughts, actions, feelings, and desires. Without regulation effort a person would respond to a situation according to habit, previous learning history, innate tendencies, or biological needs. Self-regulation is comprised of 3 sub-processes: (1) self-observation, (2) self-evaluation, and (3) self-reaction. These processes are interdependent and take place in an ongoing circular process [55]. As they are interdependent, all 3 should be reflected in the performance objectives and targeted by the intervention.

Self-observation, or self-monitoring, refers to conscious efforts to explicitly identify one’s own impulses. It is important to observe the thoughts, feelings, or environmental factors that precede craving or deciding to have a drink (eg, “the argument with my spouse made me feel poorly, and I had a drink to cheer me up” or “meeting my friend at the pub made me feel like drinking”). Self-evaluation involves using criteria or standards to assess situations, problems, or behaviors according to personal goals (eg, “my goal today was maximum 2 beers, but now I’ve emptied the fourth bottle - this was not what I planned for”). Self-reaction refers to any active effort to alter an unwanted impulse and comes as a response to self-observation and self-evaluation. Self-reaction includes self-stopping, rewarding or punishing oneself, making implementation intentions, coping, and action plans [56,57] “The next time someone offers me a drink I will reply, ‘no thanks’, even if I really want one”, is an example of a self-reaction that involves making both an action and a coping plan.

The decisions and processes involved when initiating a behavior change attempt may be different from those involved in maintaining a new behavior [58,59]. Thus, in terms of performance objectives it is useful to distinguish between implementing the change attempt and maintaining the change attempt. A meta-analysis suggests that relapse prevention is particularly effective for alcohol treatment [60]. Moreover, experiencing setbacks during attempts to change habits seems to be the rule rather than the exception. If not coped with adaptively, they may lead to impaired self-regulation [55,61,62]. Setbacks include lapse and relapse, and drinking more than a predefined target is a characteristic of both. The small but important difference between them is the cognitive interpretation of the situation. Specifically, when the person falls short of meeting the predefined target but keeps his/her resolve and continues the change attempt, it is a lapse, and when the person gives up the efforts to change and returns to the previous pattern, it is a relapse [63,64]. While avoiding lapses is a primary goal, one must cope adaptively with them when they occur. It is important that the person does not give up, but instead continues their efforts to change habits. Within Balance, these theoretical insights were translated into two separate performance objectives: (1) avoid lapse by coping adaptively with the antecedents of drinking, and (2) avoid relapse by resuming efforts to change following a lapse.

Self-regulatory processes place demands on people’s mental capabilities and when these mental resources are depleted, people are vulnerable to self-regulatory failure. Such failures, like a lapse or a relapse, are more likely to occur when people are tired or experience negative emotions [64-66]. Relatedly, enhancing positive affect can build resilience and help maintain behavior change attempts over time [67-71]. What is more, people drink to regulate emotions. Alcohol is often consumed in hopes to alleviate negative or enhance positive feelings and may thus be regarded a mood regulation strategy. This is an inexpedient strategy for the at-risk drinkers and they need help to discover more appropriate tools. Constructive regulation of own emotions was thus included as a third performance objective within Balance. In summary, to reach the overarching goal of the intervention program, clients must complete the performance objectives outlined in the first column of Table 1. This set of performance objectives is what we refer to when we say that the overarching idea of the treatment is to support continued self-regulation throughout the behavior change process.

#### Defining the Change Objectives of the Program

To develop the change objectives of the program, 2 considerations were made. First, what the clients needed to do to successfully change their behavior was defined (ie, the performance objectives above). Second, what makes people carry out these actions was identified (ie, the behavior determinants). For this purpose, the change objectives related to knowledge, attitudes, norms, planning, self-efficacy, skills, and behavior were included [56,57,72-76]. The performance objectives, determinants, and change objectives are displayed in Table 1. Each change objective is operationalized such that it is measureable within an evaluation, and phrased as a response to the question, “What needs to change related to the determinant for the program participants to do the performance objective?” [1]. The change objectives can be considered the basic building blocks of the behavior change process, whereas the performance objectives together with the determinants provides the overall plan of the process. Each of these three core concepts represents different levels of abstraction in the planning, and they are based on different sets of theory and evidence. Together, the elements of the matrix in Table 1 constitute a logic model for the change process that the intervention is expected to bring about. The matrix can be looked upon as a map of the active ingredients of the intervention, and constitutes the core of the treatment rationale that underpins the current intervention.

**Table 1:** The matrix of change objectives.

Performance objectives for at-risk drinkers	Determinants		
	Knowledge and outcome expectancies	Attitudes and self-efficacy	Planning, skills, and actions
1. Continued self-observation and self-evaluation	Express that sustained effort in self-observation/ evaluation is necessary	Active involvement in own change attempt Express positive attitudes towards behavior change (eg, it is interesting, entertaining, instructive) Express confidence in ability to observe and evaluate self	Keep a record of drinks and compare with personal standard Adjust the maximum limits for consumption according to recent experience Be able to detect the antecedents of drinking
2. Implementation of the behavior change attempt	Know how own drinking relates to official guidelines	Express positive feelings for receiving help to drink less Express confidence in ability to implement change	Set exact maximum limits for the number of drinks to be consumed Make informed choice of whether to drink less or not
3. Maintenance of the behavior change attempt over time	Recognize relapse vulnerability and need for long-term efforts	Express that intervention provides help that are personally relevant and according to own goals Express confidence in ability to maintain change.	Plan how and when to reward oneself for achievements Log on to the program regularly Activate support from the environment
3a. Avoid lapse by coping adaptively with the antecedents of drinking	List the most personally relevant antecedents of drinking	Express confidence in ability to cope with urges and temptations etc	Make implementation intentions about activating tools and strategies to handle craving or temptation, including techniques to improve mood
3b. Avoid relapse by resuming the change effort after a lapse	Know the psychological consequences of having a lapse and the distinction between lapse and relapse	Attribute failures to transient situational factors and achievements to self Express importance of achievements and downplay setbacks State that starting to drink more after a lapse is a deliberate choice Express confidence in ability to recover after a lapse	Make implementation intention, after a lapse, about sticking to original plan (drink less)
3c. Constructive regulation of emotions	List a set of techniques to improve mood	Express confidence in ability to regulate mood	Apply the learned mood regulation techniques

### Theory Informed Methods and Practical Applications

The previous step of the mapping process is largely concerned with what needs to change (ie, conceptual theories), while the current step is concerned with how change is brought about (ie, action theories). As demonstrated in the previous step, self-regulation theory [55] was essential to the treatment rationale, in that the self-regulation perspective was used to develop the performance objectives. The performance objectives are in turn used to deduce the more specific change objectives and to select methods and practical applications that address these change objectives. In this way the performance objectives, and thereby the self-regulation perspective, guided how additional theories and models were included in the treatment rationale. Specifically, cognitive behavior therapy [74] and the transtheoretical model [76] describe several methods to stimulate the 3 basic sub-processes of self-regulation, self-observation, self-evaluation, and self-reaction, respectively. These methods include raising awareness, psychoeducation, dramatic relief, self-reevaluation, environmental reevaluation, modeling, relaxation training, training in problem solving skills, reinforcement, and feedback [74,76]. The Health Action Process Approach [77] was used to distinguish between pre-action self-efficacy, maintenance self-efficacy, and recovery self-efficacy. This means that the self-efficacy should be targeted in relation to important behavior change challenges like the abilities to maintain the change attempt and recover from setbacks. Stated differently, self-efficacy needs to be targeted in relation to each performance objective. Social cognitive theory was used to specify 3 sources of self-efficacy: overt mastery experiences, vicarious experiences, and verbal persuasion that each provides specific targets for intervention [73].

Making heavy drinkers track their own alcohol consumption on a daily basis using automated technology may help to reduce drinking [78]. Also, to set specific personal goals for themselves with regard to lowering consumption is a recommended method for drinking with moderation [79]. Setting specific goals in combination with behavioral monitoring allows for comparison of goals with actual behavior, which is a crucial ingredient of the self-regulation process (self-evaluation). Moreover, goal setting combined with behavioral monitoring may serve as a trigger system for launching a just-in-time relapse prevention therapy [53]. Planning and forming implementation intentions are also promising methods to reduce drinking [57,80]. Positive psychology, in addition to cognitive behavior therapy [74], was instrumental in informing the design team on methods for emotion regulation [81-84], relevant to the last performance objective. Giving the program human like features, or making it person-like (ie, personification of the program) can increase persuasiveness [85]. Last but not least, inspiration was drawn from the principles of motivational interviewing, including express empathy, develop discrepancy, avoid argumentation, roll with resistance, support self-efficacy, and emphasize client autonomy [73,86,87]. Taken together, the principles of motivational interviewing and of persuasive technology, and the elements from positive psychology, are important in building client confidence in the program, and fostering therapeutic alliance [70,88].

In translating methods into practical strategies, one needs to consider the feasibility and the practical context. Thus, this task has to be done in iterative steps with the next task, developing the actual materials, to fit the strategies with this context. For example, the combination of goal setting and behavioral monitoring to serve as a trigger system for launching the just-in-time therapy would not be feasible in the practical context of group therapy with biweekly meetings. Table 2 gives an overview of selected theoretical methods, practical strategies, and considerations for use. These considerations are the conditions under which the methods are believed to be effective. They can be drawn from theory, empirical findings, or could be practical concerns, and they are essential to keep in mind when translating methods into practical strategies and program components.

**Table 2:** Theoretical methods, practical strategies, and considerations for use.

Theoretical method	Practical strategy: What should be done?	Considerations for use: How should it be done?
Active learning	Give information in texts (a psychoeducational approach). Cognitive and behavioral assignments. Quiz for repetition purposes.	Should be relevant, plain, rewarding to follow, and vary in format and media. Learning moments should be short and many, rather than few and lengthy.
Consciousness raising	Provide information, guidelines, assignments, examples and tips to increase self-awareness.	Feedback and confrontation should be followed by increase in problem solving ability and self-efficacy.
Self-reward	Encourage self-reward.	Should be a clear criterion for acquiring a pre specified reward.
Reattribution	Teach to explain setbacks and successes in terms of adaptive attributions (ie, transient and external attributions for failure, and stable and internal attributions for mastery).	Optimistic attribution pattern should be primed early, and reinforced after lapse (just-in-time).
Provide social cues	Provide social cues (physical, psychological, language, social dynamics, and social roles) that elicit instinctive social responses.	Excessive use of these techniques may backfire into annoyance.
Visible expectations	Stimulate thinking about expectations from significant others.	Timing: prior to drinking situations, weekends.
Self-reevaluation	Further cognitive and affective assessments of one’s self-image with and without at-risk drinking (eg, comparing self-image of being at-risk versus no-risk).	Raising awareness must be quickly followed by increase in problem solving ability and self-efficacy.
Environmental reevaluation	Further affective and cognitive assessments of how the presence or absence of risky drinking affects one’s social environment (eg, describes how drinking affects family and reflect on self as role model).	Raising awareness must be quickly followed by increase in problem solving ability and self-efficacy.
Anticipated regret	Stimulate anticipation the negative affective consequences of continued at-risk drinking.	Must stimulate imagination.
Modeling	Show potential role models and how they coped with difficulties etc.	Model should be reinforced.
Resistance to pressure	Promote making of counter arguments.	Requires building of refusal skills.
Positive self-talk	Encourage making positive statements to inner ear about self, own abilities etc.	Not applicable.
Reframing	Teach how to put negative facts into another frame of reference that makes the fact positive or neutral.	Not applicable.
Support	Stimulate mapping the environment for potential supporters. Encourage contact, and provide suggestion for contact email.	Not applicable.
Implementation intentions	Stimulate formation of implementation intentions, by texts, prompts and assignments.	Must include specification of when, where and how to act.
Planning coping responses	Promote identification of potential barriers and ways to overcome these.	Not applicable.
Mastery experiences	Teach to imagine and write down previous mastery experiences, and encourage a focus on what is mastered until now (eg, you have kept your targets for many days).	Beneficial with domain similarity. Can be used for just-in-time therapy in critical situations.
Vicarious experience	Provide stories of mastery/success from others, and encourage identification of such stories in own environment.	Requires identification with model.
Persuasion	Communicate optimism about the outcomes, and point out that change is not an instantaneous venture.	Enhanced by the prior development of confidence in treatment provider.
Behavioral monitoring	Prompt to perform daily logging of target behavior.	Not applicable.
Goal setting	Encourage setting specific and time-targeted goals with regard to drinking.	Not applicable.
Count the good things in life	Promote noticing and appreciating the positive aspects of life—anticipate future pleasures, mindful of present pleasures, and reminisce about past pleasures.	Not applicable.
Socializing	Encourage mapping social network for doing pleasant activities. Encourage contact, and provide suggestion for contact text messages or phone calls. Tips to make or improve social bonds.	Persons should decide in advance not to drink, go to places without alcohol, or with persons that do not drink.
Cognitive defusion	Encourage combating the tendency to reify thoughts, emotions, and memories.	Acceptance and defusion is not an end in itself, but a mean to increase psychological flexibility and value based action.
Mindfulness	Provide exercises that fosters contact with the present moment and self as a context, not self as the content of thoughts.	Not applicable.
Identify value-based goals	Promote defining core values, deciding specific value based goals, and acting on the goals.	Goals should be specific, measurable, achievable, realistic and time-targeted.
Nonviolent communication	Teach to distinguish an action from the assessment of or the feelings evoked by the action, identifying and expressing the feeling, the need and what one want in a non-demanding way.	Client should practice the distinctions and the concept and be given feedback.
Doing kind acts	Encourage ideas for kind acts, keep track of them, and plan them ahead of time.	Not applicable.
Visualizing best possible life	Encourage envisioning scenarios of a future life in which many goals and dreams are actualized and personal potential had been met.	Recognize what is already achieved, challenge barrier thoughts, and break major goals down into achievable sub-goals and milestones.

### Program Components and Materials

#### Screening

Screening may support an informed choice about whether to change drinking habits or not, and starting alcohol interventions with screening is standard practice [21,28,38]. Screening is also important in that it serves as a vehicle for recruiting persons from the target group to a more comprehensive treatment program. The current intervention is therefore initiated with a screening procedure, based on the Fast Alcohol Screening Test [89]. During the screening, the person receives brief individualized feedback, comparing the reported alcohol habits with health authority recommendations. After the screening and feedback, the at-risk drinkers (those with a Fast-score of 3 or higher) are recommended they sign up for the comprehensive follow-up intervention.

#### Day-to-Day Tunnel Design and Program Structure

The program relies on 2 distinct and complementary strategies for delivering intervention content—tunnel information architecture and just-in-time therapy. The tunnel information architecture is a core organizing feature of the program. Tunnel designs use a screen-by-screen and a session-by-session approach in which the user follows a predetermined sequence of units of content. As opposed to in a hierarchical design, where the user must navigate menus to find the desired content, the user is guided through the various program materials in a fixed sequence in a tunnel design. To avoid distraction, a tunnel program restricts access to any ancillary or related content, and oftentimes it limits user navigation to the “next” and “prior” buttons, thus offering low user workload [90].

A tunnel design is double-edged sword. Upon entering a tunnel the user accepts a lowered degree of autonomy, and there is a danger that reduced autonomy may lead to frustration and dropout. On the other hand, a tunnel design may also increase the chances that the user will engage in activities and see content that would otherwise not be encountered [85]. A recent randomized trial [91] compared a tunnel design to a high user control version of the same intervention content. Subjects in the tunnel design condition perceived the efficiency of the intervention to be lower, compared to subjects in the freedom of choice condition. In terms of outcome, however, the tunnel design was found to increase the number of screens visited and knowledge gained from the eHealth intervention, compared to the freedom of choice condition. In other words, there was discordance between user preference and the actual outcome. This trial demonstrates that although users seem to prefer freedom of choice, restricting this choice by using a tunnel design, can in some cases more than compensate for the disadvantages of a tunnel design. Also, two trials of a smoking cessation intervention that used a tunnel design similar to the current one showed high program adherence and efficacy [54,92,93], thus demonstrating the feasibility of the tunnel design to behavior change and adding to the promise of a tunnel approach.

In the current program, the tunnel design was applied both at micro and macro levels. On a micro level, it dictated that each session was broken into smaller portions or pages; the user can flip between pages but not sessions (Multimedia Appendix 1). On a macro level, it meant there was a fixed day-to-day sequence of sessions that require users to go through the sessions in the predetermined sequence. A session that is accessed on one day cannot be accessed the day after. If the client does not log onto the site for a period, however, he or she can catch up by accessing the preceding, unread sessions. This tunnel design is in accordance with the need for continuous self-regulation, and in line with the recommendation that self-help alcohol interventions should provide support for a minimum of 6 weeks [94]. The distributed and frequent contact points stimulate awareness of one’s own change attempts, serve as frequent prompts for self-regulation, and represent a tacit way of telling the clients that behavior change is a process that call for sustained effort.

Including proactive elements and supplementary modes of communication can improve adherence to Web-based interventions [12,95,96]. Hence, for each session the client is sent a reminder email that contains a link for the session of that day. During the active behavior change phase, which is the first 2 months of the program, the client is given access to a new and unique session each day. This is followed by a low-intensity maintenance phase. Here, the numbers of sessions are reduced, first to 1 per week for the first month, then to every second week for the next month, and finally, to once every month for 8 months. In total, Balance comprises 73 sessions. An average session typically consists of 1000 words, split between 10 to 15 screens. The language is categorized as the easiest out of 6 levels on the LIX readability measure (ie, “very easy to read, equivalent to juvenile books”) [97]. Each session takes from 3 to 10 minutes to complete, depending on the clients’ depth of processing and speed of reading.

Although a tunnel design restricts user self-determination, such a design does not dictate passive users, as the tunnel design is well suited to foster interactive dialogues with the user [90]. Hence, the sessions include interactive tasks and cognitive behavioral assignments. For example, each week there is a quiz, consisting of 5 multiple choice questions, pertaining to the most important learning points of that week. Along with a brief summary statement of the learning point, the client gets immediate feedback on whether the response is correct or not for each question (Multimedia Appendix 2). Moreover, cognitive behavioral assignments that the clients are supposed to do between sessions can be provided in one session and then the assignment can be elaborated on during the next session. An example of a cognitive task to support self-regulation, used in the current intervention, is to make up and write implementation intentions of what to do in a tempting situation, and then later the client is later asked to adjust and improve this list according to recent experience.

#### Goal Setting, Behavior Logging, and Just-in-Time Therapy

In the current intervention, goal setting, behavior logging, and the just-in-time therapy are practically and theoretically intertwined. Each session the client is asked to log the number of drinks had on the previous day. Such behavior logging is important because it stimulates self-awareness. During each Monday session, the client determines their drinking targets for the coming week. To support this goal setting, a graph presenting the targets set and the logged consumption (week totals from all previous weeks in the program), and a detailed comparison of target and result for each day from the last week, is displayed. Then the client is asked whether the goals from previous week should be kept or adjusted. If they choose to adjust the targets, they are provided with a form where they fill in the maximum number of drinks to be consumed for each day of the week. The client is encouraged to cut down slowly at first, and then gradually cut further down as the initial targets are met. The client is told to set targets that he/she perceives to be achievable. By making the clients set their own targets, client autonomy is maintained. However, if the week goal is higher than baseline, this is pointed out to the client and the client is asked whether he/she is sure about the targets. The client may either respond “Yes, this week is special and I want to allow myself such goals”, or “No, I want to set myself lower goals.” As such, behavior logging and goal setting may contribute to successful behavior change [78,79]. These elements are essential within Balance because they are used as part of a trigger system for providing 2 instances of just-in-time therapy—a relapse prevention system and a lapse prevention system.

#### Relapse Prevention System

After logging of the drinks consumed on the previous day, the datum is compared with the target that was previously set by the client for that day (Multimedia Appendix 1). This happens in every session. If the result is below or on target, the client is praised, for example, “Well done! You drank less than your goal yesterday,” or “Great! You did well yesterday. You are right on target!” However, if a client drank above target, he/she is asked: “What happened yesterday? Was it just a slip-up or do you think you are about to fail?” The clients are taught the distinction between lapse and relapse earlier in the intervention, and therefore can make the distinction between a slip-up and failure. If the client reports that it was “just a slip-up” or a lapse, the client can simply move on to the session. If not, then 1 of 3 relapse prevention therapies is launched. The system will remember previous lapses, so that each client will not receive the same therapy twice before the fourth reported lapse. In this way, the goal setting and behavior logging procedures taking place in a day-to-day tunnel design provide a platform from which the just-in-time therapy is launched.

The therapy consists of a prerecorded dialogue between a client and a counselor (a 5 minute audio recording, Multimedia Appendix 3), as well as 3 text screens (Multimedia Appendix 1). This therapy has several purposes including increasing self-efficacy by making the client realize what is achieved up until now [73] to avoid attributing the lapse to internal and stable factors, and instead attributing the lapse to situational factors to prevent negative emotions and a full-blown relapse [61]. Finally and most importantly, the client should recognize that if he/she relapses, it is part of a deliberate decision and not something he/she is powerless in preventing.

#### Lapse Prevention System

During each session, the lapse prevention system follows the relapse prevention described above. Here, the client is reminded of that day’s target and asked how confident he/she feels about reaching it. The lapse prevention therapy is activated if the client replies, “I'm not sure” (as opposed to, “I'm fairly certain I’ll manage”). Before the therapy starts, the client is asked to elaborate, by selecting 1 of 3 options: (1) “I feel worn out and down”, (2) “I don’t feel so sure of myself today,” and (3) “I seem to have lost some motivation.” The options are intended to cover depletion of resources, self-efficacy, and motivation, respectively (Multimedia Appendix 1).

At the final screen of the lapse prevention therapy, the client is asked to provide a time point for that day or evening for the program to send an encouraging mobile phone message via short message service (SMS). The client is encouraged to provide a time point when they would need it the most. The content of the SMS is related to the topic of the lapse prevention therapy. For example, if the therapy included planning how to cope with a challenging situation, the SMS would be a reminder about the coping plan; if the topic is previous mastery experiences, the SMS would be a reminder of those previous experiences.

#### Personification

People tend to react to and interact with objects in their environment, including media applications, as if it were real people [98]. It is assumed that mimicking features from human-to-human interaction in human computer-based communication can foster a sense of relationship or alliance between the program and user. This may in turn increase program impact [71,85,88,96,99]. Each session thus starts with a greeting and ends with a “goodbye”, as if Balance is a person (Multimedia Appendix 1). The language is informal in tone, and oftentimes, personal pronouns are used. Additionally, pictures of 3 different guides or coaches that each represent their special topic, embodies or personifies the intervention. For example, each time the topic has to do with emotion, the client sees a picture of the “mood coach” along with the text. The 2 other guides are the motivation and willpower coaches. The idea behind such personification of the program is that humans are predisposed to respond to cues that they can easily connect with. Pictures and language (praising, greetings, turn-taking, interactivity) provide social cues that may in turn elicit the corresponding social responses from the client [85]. Speaking directly to patients about their patient status or the working alliance, however, was avoided, as this can be counterproductive and cause resistance [100].

#### Emotion Regulation Components

Emotion regulation makes up a significant proportion of the intervention content, and consists of 7 distinct tracks, each covering a unique topic including gratitude, socializing, turning negative thoughts, nonviolent communication, doing acts of kindness, optimism, and pleasant activities. The contents and assignments from these tracks are taken from the positive psychology tradition and from cognitive behavioral therapy [81,82,101-103]. Each user, however, follows only 4 of the 7 available tracks. Each week during the active behavior change phase, emotion regulation is targeted in 4 of the 7 sessions. This means that all 4 topics are visited once weekly during the initial 2 months of the program.

Two of the tracks are provided to all users, the gratitude track and the socializing track. The gratitude track involves assignments like counting ones blessings, writing down 3 good things that happened during the day, writing a letter of gratitude, and saying “thank you” more often than usual. The socializing track is about encouraging social interaction with friends and relatives (without alcohol). Assignments in this track includes calling a friend and inviting him/her out, mapping one’s social network, advice on how to get new friends, and strengthening existing relationships (eg, find out about a friend’s plan and follow up the next week by asking how it went, or give compliments to a partner or friend).

Then, based on a cue-reactivity test [104], designed to distinguish those mainly triggered by conflict situations from those triggered mostly by negative emotions and pain [61], users get either the turning negative thoughts or nonviolent communication tracks. In the test, clients are briefly shown a drawing depicting a potential trigger situation for drinking, and are subsequently asked to what extent they would feel able to resist having a drink after such a situation. The drawings incorporate a title word describing the potential trigger situation. For conflict situations, the picture headings include confrontation, aggression, fight, and criticism, while the negative emotion drawings include guilt, sadness, sickness, and stress. Those primarily triggered by conflict situations are recommended to follow the nonviolent communication track, while those triggered by negative emotional states are recommended to follow the turning negative thoughts track. To preserve client autonomy, however, the client is given the option of overruling the recommendations and select tracks regardless of test results.

The turning negative thoughts track is based on the acceptance and commitment therapy [105] and uses a combination of acceptance and mindfulness to help people distinguish themselves from thoughts, feelings, sensations, and memories. It also clarifies their personal values and take action based on those. This track includes a cognitive defusion technique called the word repeating technique, and a mindfulness exercise called “take your mind for a walk”. In the mindful exercise, the client envisions a mental journey through a forest, and each time a thought that is irrelevant to the forest comes up, the client is instructed to visualize putting the thought under a stone and keep on walking. Additionally, clients are encouraged to define their values, rank them in importance, and set concrete goals for the most important ones. The goal of this track is to help turn around negative thinking, and thereby reduce the likelihood of lapse or relapse. Nonviolent communication serves as a basis for another track [106]. Here, empathy and honest self-expression is emphasized. First, the client is trained to distinguish an action from the assessment of, or the feelings evoked by, the action. Second, the client identifies and expresses the feeling. Third, the client identifies and expresses the need. Last, the client expresses the need in a non-demanding way. The goal of this track is to help clients reduce the level of conflict in their everyday interactions, and hence reduce the likelihood of relapse.

Then based on a person-activity fit diagnostic [107], the client is recommended to choose 1 of 3 tracks—acts of kindness, optimism, and pleasant activities. Again, to preserve client autonomy, he or she is given the option of overruling the recommendations and select tracks regardless of test results. In the acts of kindness track, clients are encouraged to do acts of kindness to others, keeping track of such acts, and plan them ahead of time. Examples of kind acts are provided, and clients are encouraged to figure out such acts for themselves. The track targeting optimism is based on “the best possible self” exercise taken from positive psychology [82,108]. Here, the client is encouraged to envision scenarios of a future life in which many goals and dreams had been actualized and where much personal potential had been met. Assignments in the coming week will ask him or her to elaborate on the scenarios, to recognize what they have already achieved is in line with the best possible life scenario, challenge and turn around obstructive thoughts, and break major goals down into achievable sub-goals and milestones. The pleasant activities track is based on cognitive behavioral therapy [103], and starts by prompting the client to compile a list of activities that make him/her feel good. In the subsequent weeks, the client is asked to schedule one or more of these activities. He or she is also asked to determine a goal for one of the activities, which is something to achieve by repeating the activity over time.

## Discussion

We have used IM to give a systematic and comprehensive description of the treatment rationale of an alcohol intervention, named Balance. Its treatment rationale is essentially based on a self-regulation perspective [55,61,64], in that the overarching idea of the program is to support continued self-regulation throughout the behavior change process. Maintaining the change efforts over time and coping adaptively with challenging situations are key factors to successful behavior change [55-71,109]. In achieving this, 3 elements of the treatment rationale are of particular importance: (1) emotion regulation as an inoculation strategy against self-regulation failure, (2) avoiding lapses by adaptive coping, and (3) avoiding relapse (if lapses occur) by resuming the change efforts after a lapse. In terms of delivery, the current intervention relies on 2 distinct but complementary delivery strategies, including a day-to-day tunneled psycho-educational approach in combination with the just-in-time therapy. The tunnel design is in accordance with the need for continuous self-regulation and at the same time it functions as a platform from which the just-in-time therapy can be launched. Two instances of just-in-time therapy are included as part of an emphasis on coping during critical moments of the change process (before and after a lapse). First, a lapse prevention system, which starts when the client reports low self-efficacy, and second, a relapse prevention system that is activated when the client reports drinking above target level.

Tailoring of intervention content in response to dynamic processes in target behavior or emotional states, as described above, is an emerging trend to eHealth design [2,44-54]. This and similar forms of intervening just-in-time can potentially play a more significant role in future interventions as advances in mobile technology make it more feasible, and increasingly available at a population level [2,44,50]. More research is needed to determine how this potential is to be released. For example, we do not know which theoretical frameworks are the most appropriate to underpin such interventions. As opposed to the more common approach to tailoring interventions that is based on differences between participants at baseline, just-in-time adaptations need to lean on models that are dynamic representations of within-person processes [2]. As the self-regulation theory focuses on within-person processes in behavior change, it may be one of the feasible perspectives for this purpose. In the current paper just-in-time components was placed within a complete theoretical and practical context, including the tunnel delivery strategy and the self-regulation perspective. By describing this treatment context we have contributed to an understanding of how multiple delivery strategies in eHealth interventions can be combined—for general behavior change interventions, as well as alcohol specific interventions.

### Limitations

There are several limitations to the current research. The target group of the program is very broad. On one side of the spectrum, the target group includes persons with a long history of harmful drinking that might be very conscious about the negative impact from alcohol on their life, that are already motivated to change. On the other side of spectrum, the target group also includes persons that drink only marginally above the limits for sensible drinking. They have probably never experienced any serious negative consequences of alcohol, their drinking pattern is largely within the borders of what is culturally acceptable, and many of them probably do not think that they need to change at all. Targeting such a diverse population, with regard to motivation for change, drinking pattern, and alcohol history, with one single intervention may turn out to be overly optimistic. That is, the intervention will be more or less acceptable to use for certain sub-groups within the broader target population. Exactly what outcomes to expect in the various sub-groups, however, is an empirical question, which we will try to shed light on in later evaluation reports.

The dropout rates from eHealth interventions, including alcohol interventions, tend to be high [22,110]. For example, in a sample of problem drinkers, enrolled in a 12 session Web-based intervention, a dropout rate of 45% was reported, and the authors concluded that “the challenge of Web-based alcohol treatment programs no longer seems to be their effectiveness but keeping participants involved until the end of the treatment program” [111]. In terms of number of sessions (73) the current intervention is probably the most comprehensive of its class [20-22]. For each session added to an intervention, a further opportunity to drop out is also added. Hence, there is no reason to believe that the current intervention will be an exception from “the law of attrition” [110]. Rather than counting how many people drop out before the end of treatment, we think it is more important to focus on the number of sessions completed, and by whom (in combination with reach and effectiveness). Therefore, our conclusions differ slightly from the conclusion of Postel [111]. The criterion for success in eHealth is not to keep participants until the end of treatment, but to keep them long enough to achieve a clinically significant effect on the relevant health behavior. Furthermore, what is a sufficient dose may vary from subject to subject. For one sub-group, the screening and feedback session might be enough, while another sub-group, may benefit only if they complete 10 or more sessions. We stress that, the rate of uptake in the target group, the program adherence among users of the intervention, as well as the efficacy across sub-groups, remains open empirical questions that we aim to elucidate in subsequent research.

### Advantages to the IM Approach

EHealth is an applied science in which interventions are designed to solve specific health problems, meaning that the design process ought to be problem driven [1]. To meet a treatment or prevention challenge and solve the health problem, the main tools are theory and evidence. Theory can however be approached and applied in many different ways. The design process thus require several decisions to be made, for example, regarding which theories are needed, how many, when to use conceptual theories, when to use action theories, and if one should stick to theories that predict the health problem or focus more on evidence that accounts for behavior change. In answering these and other questions, IM offers a set of guidelines on how to approach theory and evidence, in what order to use them, and for what purpose [25]. In this way, IM allows the researcher to integrate theory and empirical evidence from multiple sources to form a single causal model (eg, Table 1), which shows how the researchers intended the intervention to work, and the hypothesized causal links of the treatment [1]. IM constitutes a multi-theoretical approach as opposed to a single-theoretical approach to intervention design that ensure a problem driven focus, necessary for the researcher to stay on track with regard to solving the health issue. However, the problem driven approach is not suitable for researchers focusing primarily on theory building and testing, only for researchers focusing on intervention building and testing [25].

The step-by-step manualized approach of IM prescribes a process to designing an intervention that functions as a useful planning tool. The manualized approach is also advantageous in that it provides a clear structure to the reporting that may aid in writing it, reading it, and in comparing the rationale across interventions. In completing the IM steps, theory and evidence is systematically linked to eHealth practice through a logical chain of decisions. Each step and decision shapes how an intervention influences its users, and by making the choices explicit, they are subjected to the test of evidence [10]. This is valuable because it extends the evidence base for subsequent intervention development. Therefore we recommend the use of a structured approach, like the IM protocol, in describing intervention rationale.

### EHealth and Intervention Reporting

Scientists, reviewers, and editors alike are inevitably influenced by the trial report conventions [112,113]. These conventions together with word limits in journals result in intervention descriptions where the main focus is to describe how the intervention was evaluated rather than describing what it is [10,13]. The focus on the evaluation also manifests in the abstract, making the evaluation visible and accessible in bibliographic databases without emphasis on the intervention rationale. This situation makes it harder for a researcher and development team to compare treatments and learn from previous development efforts. When performing literature reviews, the design rationales are often embedded within multiple sections of multiple trial reports, and it can be difficult to get a full understanding of what exactly is being evaluated. This is not a problem for simple interventions, or when testing a single theory, but for complex interventions that are problem driven and multi-theoretical in nature, it becomes an issue.

Several solutions to this issue have been suggested, including digital preservation of intervention content in a Web archive [8], publishing addendums or appendixes along with the trial reports [7], as well as reporting in online journals, which tend to have less restriction on word limits [6]. These suggestions offer more space for reporting interventions. However, descriptions in an addendum or a Web archive will not be fully targeted by the peer review, thus evading quality assurance. Additionally, all these alternatives evade the full visibility afforded by academic search engines. Stated differently, these reporting practices make the important initial stages of intervention research a backstage performance. As an alternative, we espouse the view that eHealth researchers should publish the intervention protocols separately and peer-reviewed prior to publishing their evaluation studies [6,9]. Reviews that systematically assess the link between intervention rationale and outcome [12,23,114] can play a vital role in improving eHealth practice as they potentially synthesize the experiences from the entire field. The quality of these reviews depends on the quality of the intervention descriptions they build upon [13]. We believe that the above can improve the quality of available descriptions and ease comparisons of treatment rationale across interventions. Provided that reporting of interventions is improved across research teams and that reports of intervention development are combined with findings from clinical trials, the possible scope of systematic reviews will be broadened. A broadening of scope in future systematic reviews assessing the link between intervention rationale and outcome, may ultimately guide researchers in designing interventions with improved efficacy, reach, and user acceptability.

### Conclusion

The descriptions of the treatment rationale for the alcohol intervention Balance, provides an intervention blueprint that will aid in interpreting the results from future program evaluation, it will ease comparisons of intervention rationale across interventions, and it may assist intervention development. By putting the just-in-time therapy within a complete theoretical and practical context, including the tunnel delivery strategy and the self-regulation perspective, we have contributed to an understanding of how multiple delivery strategies in eHealth interventions can be combined. Additionally, this is a call for action to improve the reporting practices within eHealth research. As one way to achieve such improvement, we advocate for using a systematic and structured approach, and for intervention reports to be published peer-reviewed and separately from evaluation reports.

## Multimedia Appendix 1

A compilation of screenshots from the Balance program.

## Multimedia Appendix 2

Sample items from the weekly quizzes.

## Multimedia Appendix 3

A transcription of one of the audio therapies for managing a recent lapse (relapse prevention).

## Abbreviations

IM: intervention mapping
SMS: short message service
