# Supplementary material for: Constructing a Theory- and Evidence-Based Treatment Rationale for Complex eHealth Interventions: Development of an Online Alcohol Intervention Using an Intervention Mapping Approach
Source: JMIR Res Protoc. 2013 Jan 23;2(1):e6. doi: 10.2196/resprot.2371 (PMC3629462; doi:10.2196/resprot.2371)
Supplement: Supplementary file 1 [file resprot_v2i1e6_app1.pdf]

Multimeadia Appendix 2 Balance screenshots

This presentation is a compilation of screenshots from the Balance program.

Some slides are single screenshots taken out of their context/sequence, while others are placed in the sequence they would appear in the program.

Changetech V2.626

https://program.changetech.no/ChangeTechF.html?Mode=Trial&P=LF8NT4&UserType=TESTER

Gmail UiO FB OBF SERAF SERAF-stip BackOffice SM W.P. TLF X-port Scopus G-Schlr Dictionary Ordnett Importert fra Interne...

balance

Settings and help

Day 0

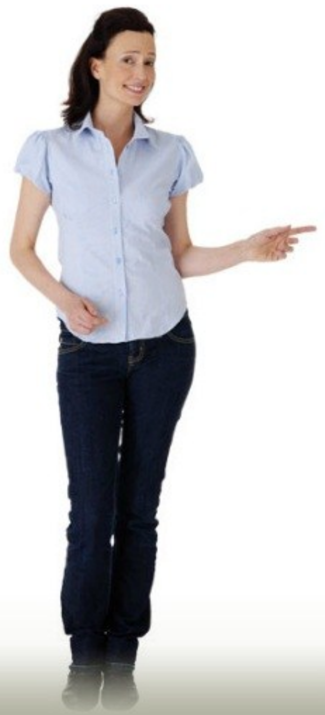

Welcome to Balance!

Wouldn't it be nice to find out a little more about your alcohol consumption? So that you can enjoy drinking without worrying about whether you "drink too much" or not.

That is what Balance is all about.

We want to help you understand your alcohol habits, not get you to stop drinking altogether. Just so that is clear.

Click here to see what Balance can do for you

Balance

NO 19:05 01.12.2012

Changetech V2.626

https://program.changetech.no/ChangeTechF.html?Mode=Trial&P=LF8NT4&UserType=TESTER

Gmail UiO FB OBF SERAF SERAF-stip BackOffice SM W.P. TLF X-port Scopus G-Schlr Dictionary Ordnett Importert fra Interne...

balance

Settings and help

Day 0

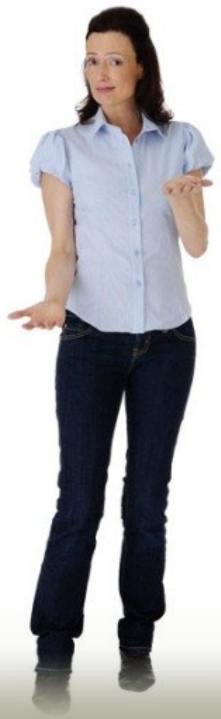

Balance consists of two parts.

First we assess your drinking habits. This assessment is anonymous, and only you get to see the results.

Perhaps there is something you would like to change? Balance can help, whether your goal is to consume less alcohol or to change the way you drink.

Everything is done online and via your mobile telephone. You do not have to attend any meetings or talk to anyone.

The Balance programme has been devised by leading researchers in the fields of psychology and behavioural change, on the basis of the latest findings from research and clinical trials.

As natural as going for a medical

Balance

NO 19:07 01.12.2012

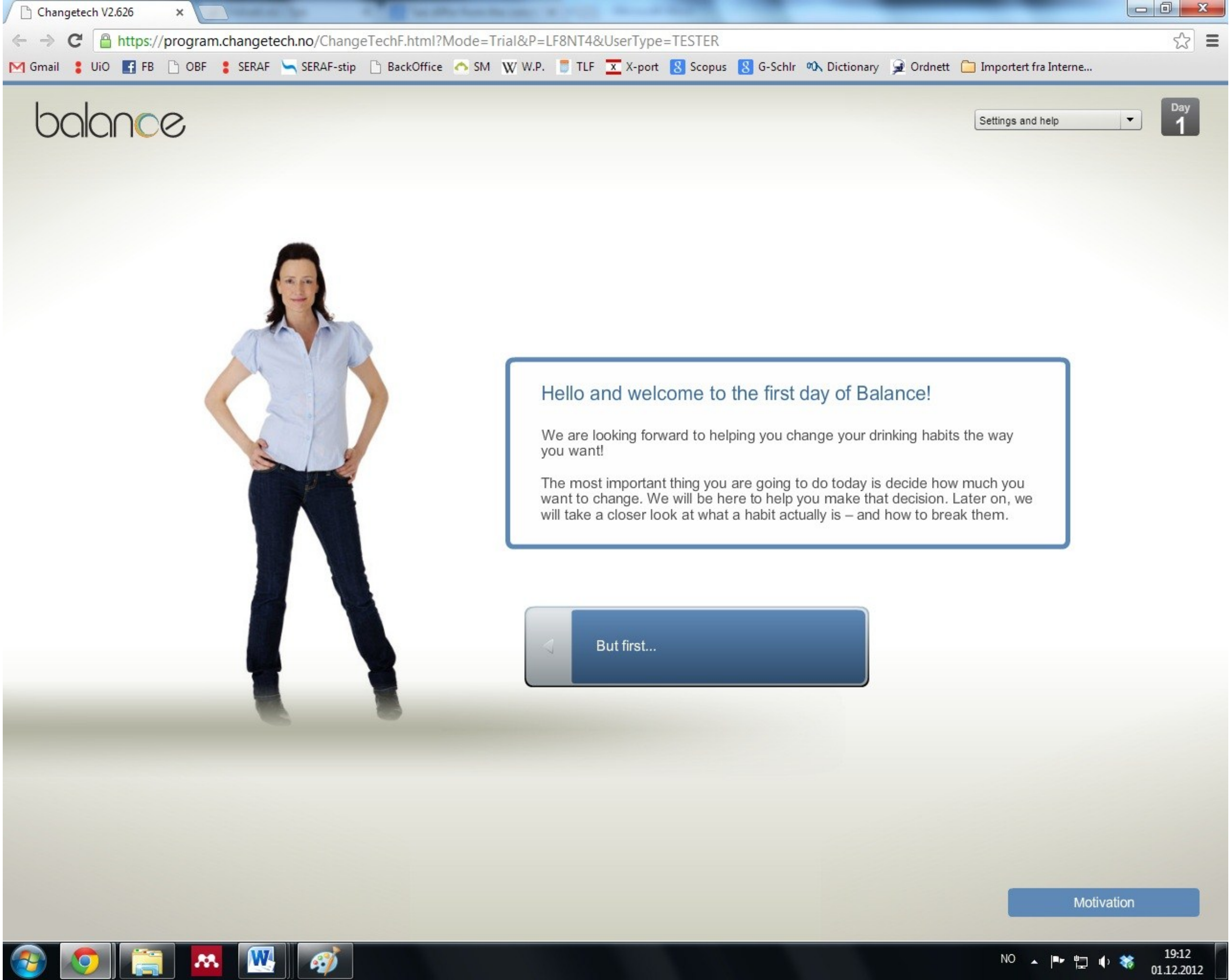
Changetech V2.626
https://program.changetech.no/ChangeTechF.html?Mode=Trial&P=LF8NT4&UserType=TESTER
Gmail UiO FB OBF SERAF SERAF-stip BackOffice SM W.P. TLF X-port Scopus G-Schlr Dictionary Ordnett Importert fra Interne...
balance
Settings and help
Day 1
Hello and welcome to the first day of Balance!
We are looking forward to helping you change your drinking habits the way you want!
The most important thing you are going to do today is decide how much you want to change. We will be here to help you make that decision. Later on, we will take a closer look at what a habit actually is – and how to break them.
But first...
Motivation
NO
19:12
01.12.2012

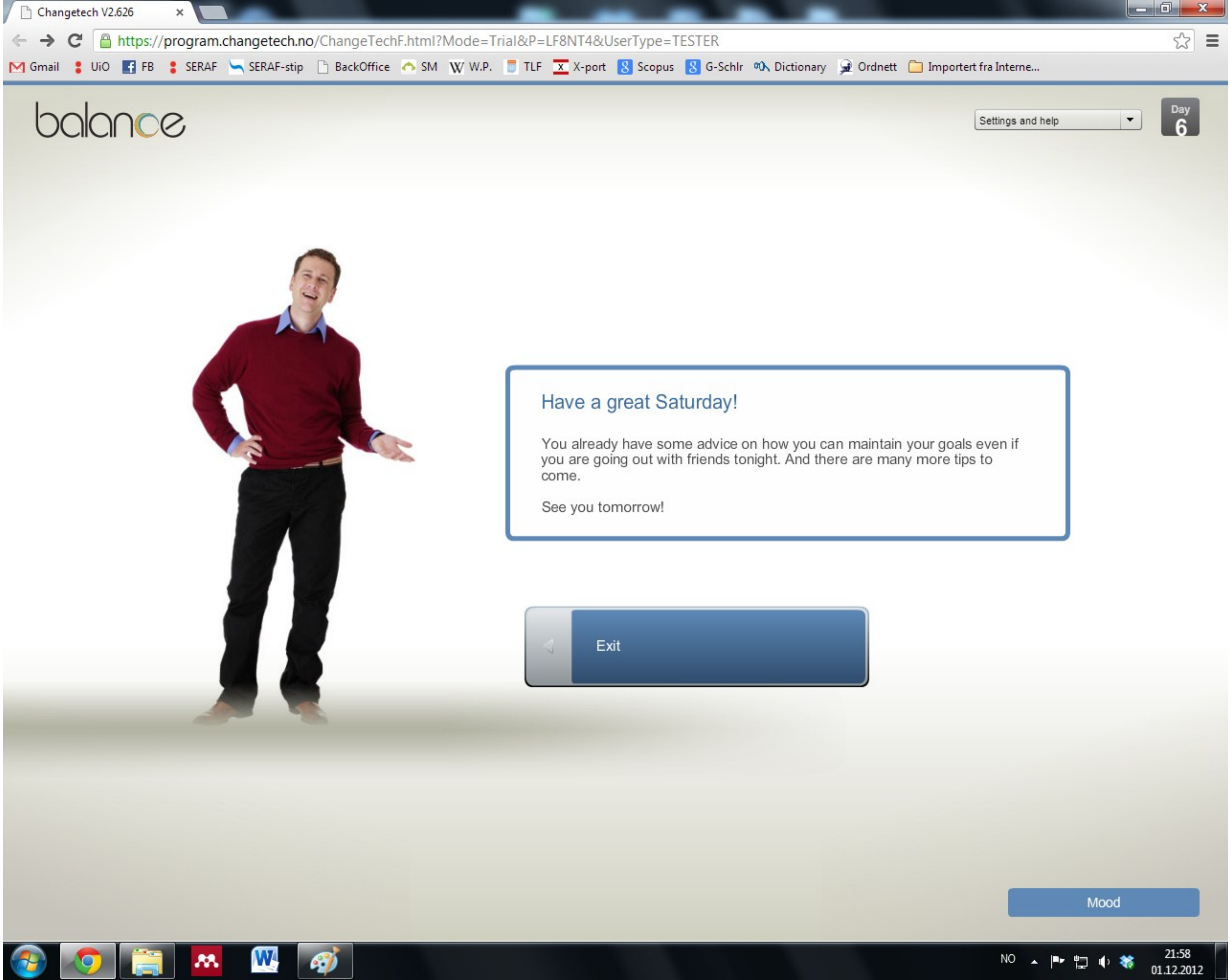
Changetech V2.626
https://program.changetech.no/ChangeTechF.html?Mode=Trial&P=LF8NT4&UserType=TESTER
Gmail UiO FB SERAF SERAF-stip BackOffice SM W.P. TLF X-port Scopus G-Schlr Dictionary Ordnett Importert fra Interne...
balance
Settings and help
Day 6
Have a great Saturday!
You already have some advice on how you can maintain your goals even if you are going out with friends tonight. And there are many more tips to come.
See you tomorrow!
Exit
Mood
NO
21:58
01.12.2012

Changetech V2.626

https://program.changetech.no/ChangeTechF.html?Mode=Trial&P=LF8NT4&UserType=TESTER

Gmail UiO FB SERAF SERAF-stip BackOffice SM W.P. TLF X-port Scopus G-Schlr Dictionary Ordnett Importert fra Interne...

balance

Settings and help

Day 7

Quiz

What do we mean by a slip-up?

- ( ) It is a relapse or regression
- (•) A slip-up means you have failed
- ( ) A slip-up is just a slip-up, and I will carry on trying

No, it doesn't. Unfortunately, we find it all too easy to think this way, but it is human to err and there is no need to make a mountain out of a molehill. Choose another option!

Continue

Motivation

NO 22:02 01.12.2012

balance

Settings and help

Day 1

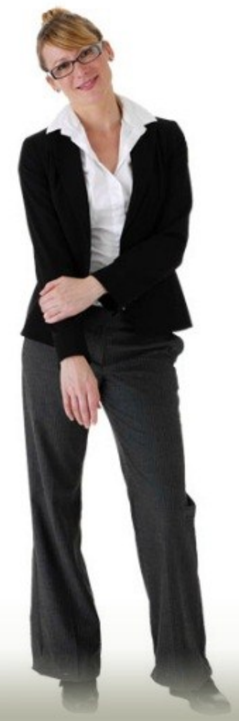

"Old habits …."

Once you have a drink in your hand, it is virtually impossible not to drink. But a lot has happened before you get to that point.

Perhaps you have come home tired, gone to the cupboard, taken out a glass, got the bottle out, poured a drink into the glass and picked up the glass, ready to drink.

That amounts to quite a few actions you have to perform before you actually have a drink. This sequence is easier to break. And the way you do it is by deciding in advance. For example: do not get the glass out of the cupboard. In the next few days, you will learn more about how to plan to break a habit.

But beware of saboteurs....

Willpower

Slip up vs. cracking

- This point is repeated across multiple sessions.
  - Note the number of the session in the right uppermost corner.

Changetech V2.626

https://program.changetech.no/ChangeTechF.html?Mode=Trial&P=LF8NT4&UserType=TESTER

balance

Settings and help

Day 2

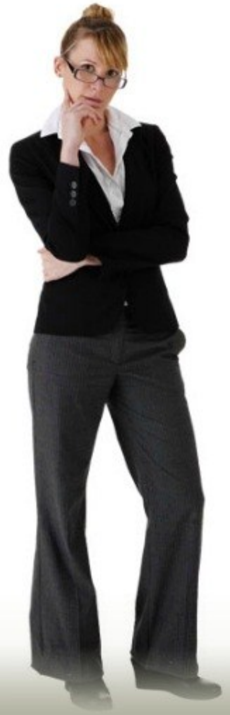

Is there a difference between slipping up and cracking?

Yes, we differentiate between the two in Balance. When you are working on changing a habit, it is normal for you to "slip up" from time to time compared with your plans.

Then, if one day you drink more than you had allocated yourself (your goal), it is easy to think:

"Damn! Now I've ruined it all. I've started drinking like I used to again. I didn't manage to change my behaviour this time (either). I might as well give up trying now and try to change later."

And then you have another glass...

But you really don't need to do this...

Willpower

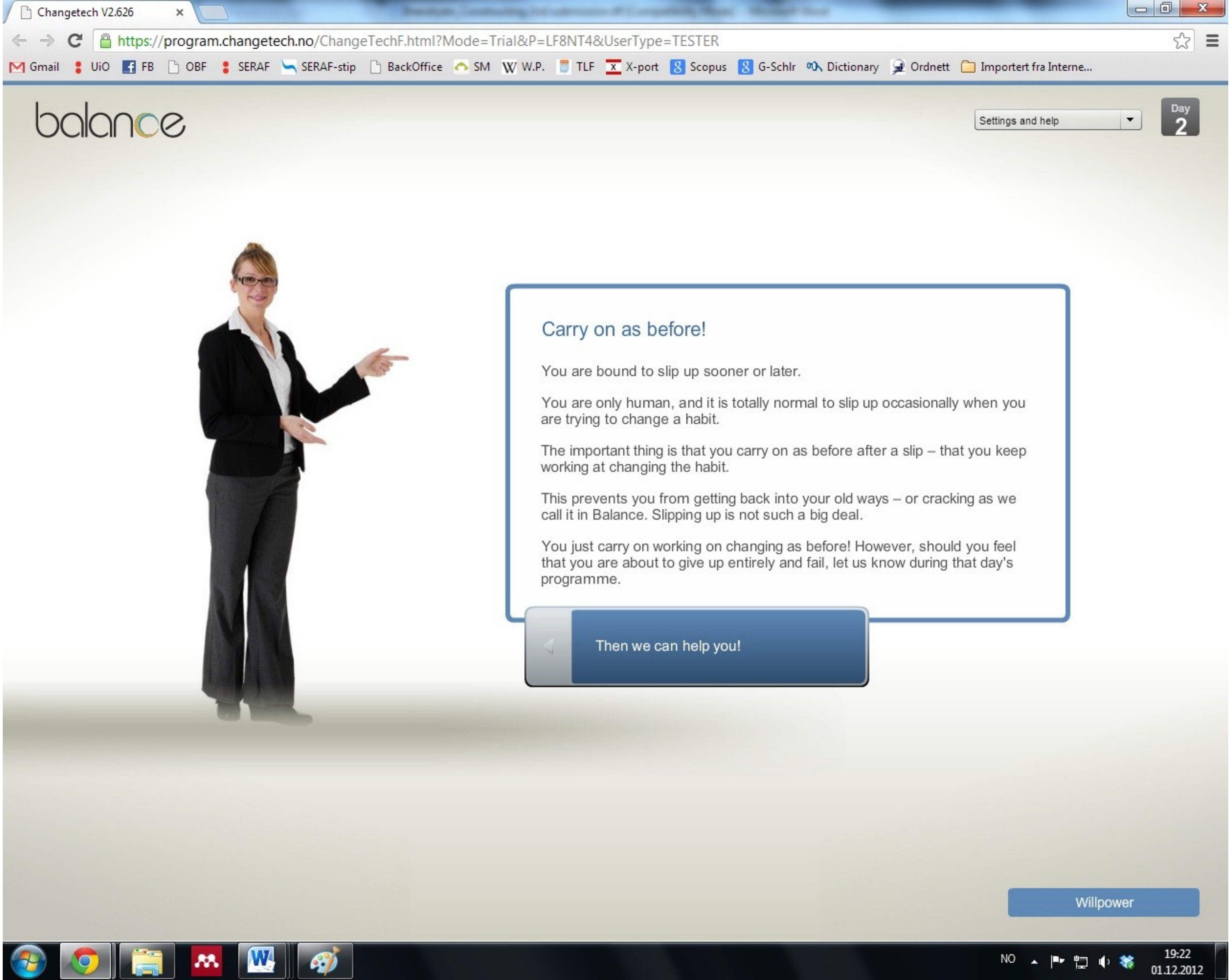
Changetech V2.626
https://program.changetech.no/ChangeTechF.html?Mode=Trial&P=LF8NT4&UserType=TESTER
Gmail UiO FB OBF SERAF SERAF-stip BackOffice SM W.P. TLF X-port Scopus G-Schlr Dictionary Ordnett Importert fra Interne...
balance
Settings and help
Day 2
Carry on as before!
You are bound to slip up sooner or later.
You are only human, and it is totally normal to slip up occasionally when you are trying to change a habit.
The important thing is that you carry on as before after a slip – that you keep working at changing the habit.
This prevents you from getting back into your old ways – or cracking as we call it in Balance. Slipping up is not such a big deal.
You just carry on working on changing as before! However, should you feel that you are about to give up entirely and fail, let us know during that day's programme.
Then we can help you!
Willpower
NO
19:22
01.12.2012

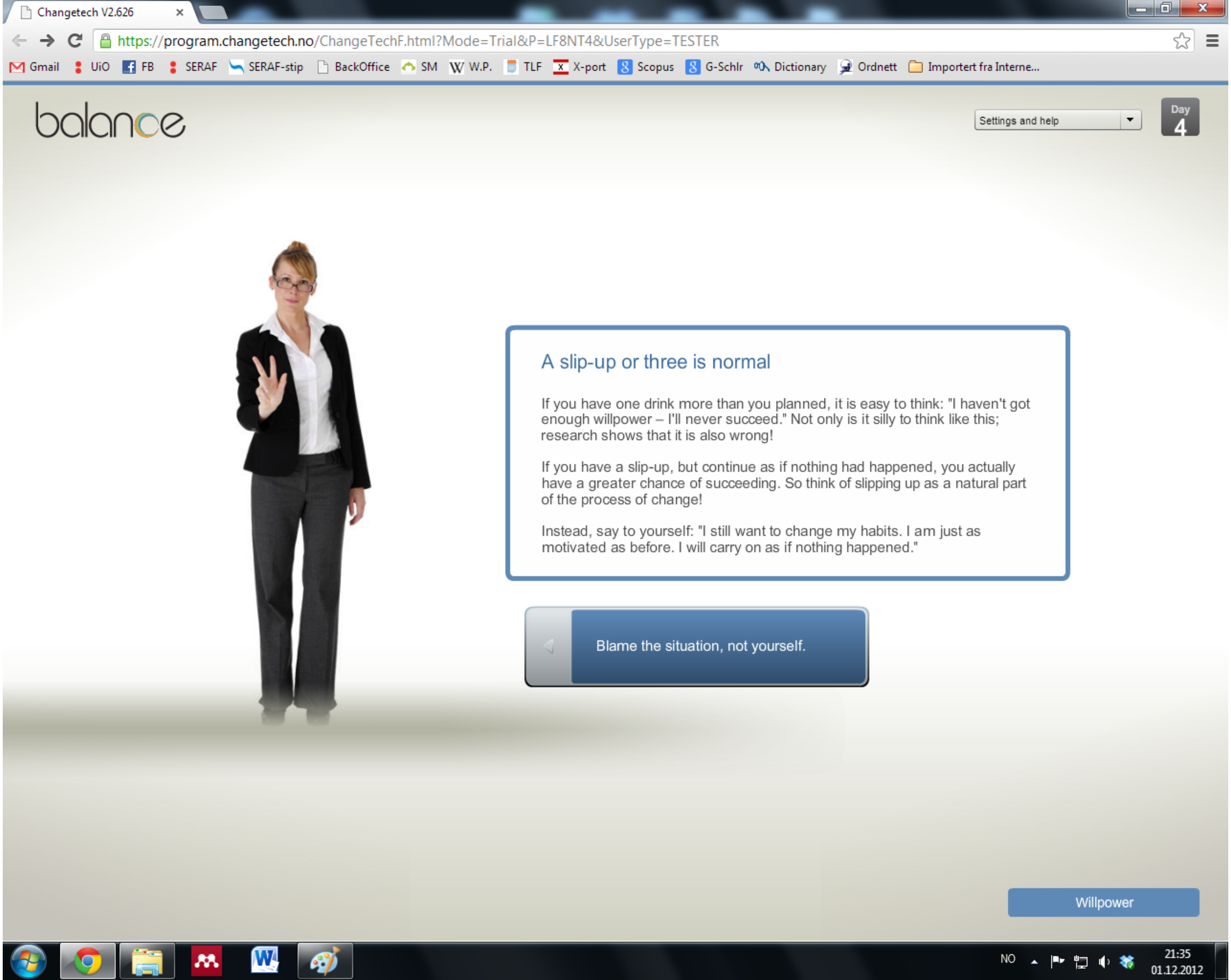
Changetech V2.626
https://program.changetech.no/ChangeTechF.html?Mode=Trial&P=LF8NT4&UserType=TESTER
Gmail UiO FB SERAF SERAF-stip BackOffice SM W.P. TLF X-port Scopus G-Schlr Dictionary Ordnett Importert fra Interne...
balance
Settings and help
Day 4
A slip-up or three is normal
If you have one drink more than you planned, it is easy to think: "I haven't got enough willpower – I'll never succeed." Not only is it silly to think like this; research shows that it is also wrong!
If you have a slip-up, but continue as if nothing had happened, you actually have a greater chance of succeeding. So think of slipping up as a natural part of the process of change!
Instead, say to yourself: "I still want to change my habits. I am just as motivated as before. I will carry on as if nothing happened."
Blame the situation, not yourself.
Willpower
NO
21:35
01.12.2012

Changetech V2.626

https://program.changetech.no/ChangeTechF.html?Mode=Trial&P=LF8NT4&UserType=TESTER

Gmail UiO FB SERAF SERAF-stip BackOffice SM W.P. TLF X-port Scopus G-Schlr Dictionary Ordnett Importert fra Interne...

balance

Settings and help

Day 4

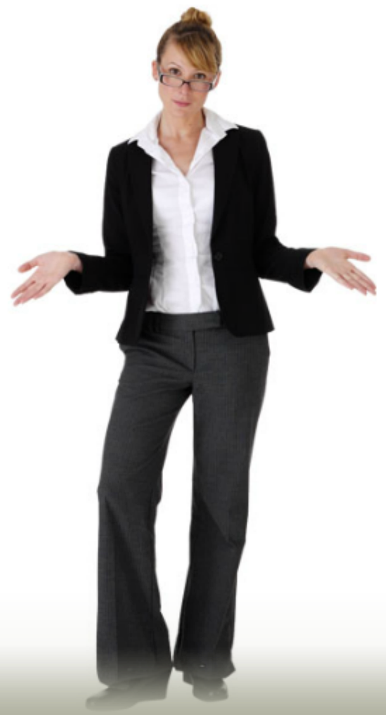

The situation was over a long time ago!

It is tempting to think: "I slipped up because I am weak-willed." Or: "I'm not good enough."

What you should really tell yourself is: "It's because I was really stressed at work that day." Or "That drink was extra tempting – after all, it was a party!"

The advantage of thinking this way is that all these types of situations do of course pass. And once the situation has passed, all you have to do is carry on working on changing your habits.

Plus, now you are one important experience richer, which will help you avoid failing (reverting to your old ways) further down the line.

So, how did you get on yesterday?

Willpower

NO 21:36 01.12.2012

Lapse- and relapse prevention

- In each session the user logs his/her alcohol consumption and self-efficacy.
  - Depending on the user input a relapse prevention therapy or a lapse prevention therapy is launched.
  - The two instances of "just-in-time therapy ".
  - The following sequence shows one example of each.
  - The user will be given a new therapy sequence each time.

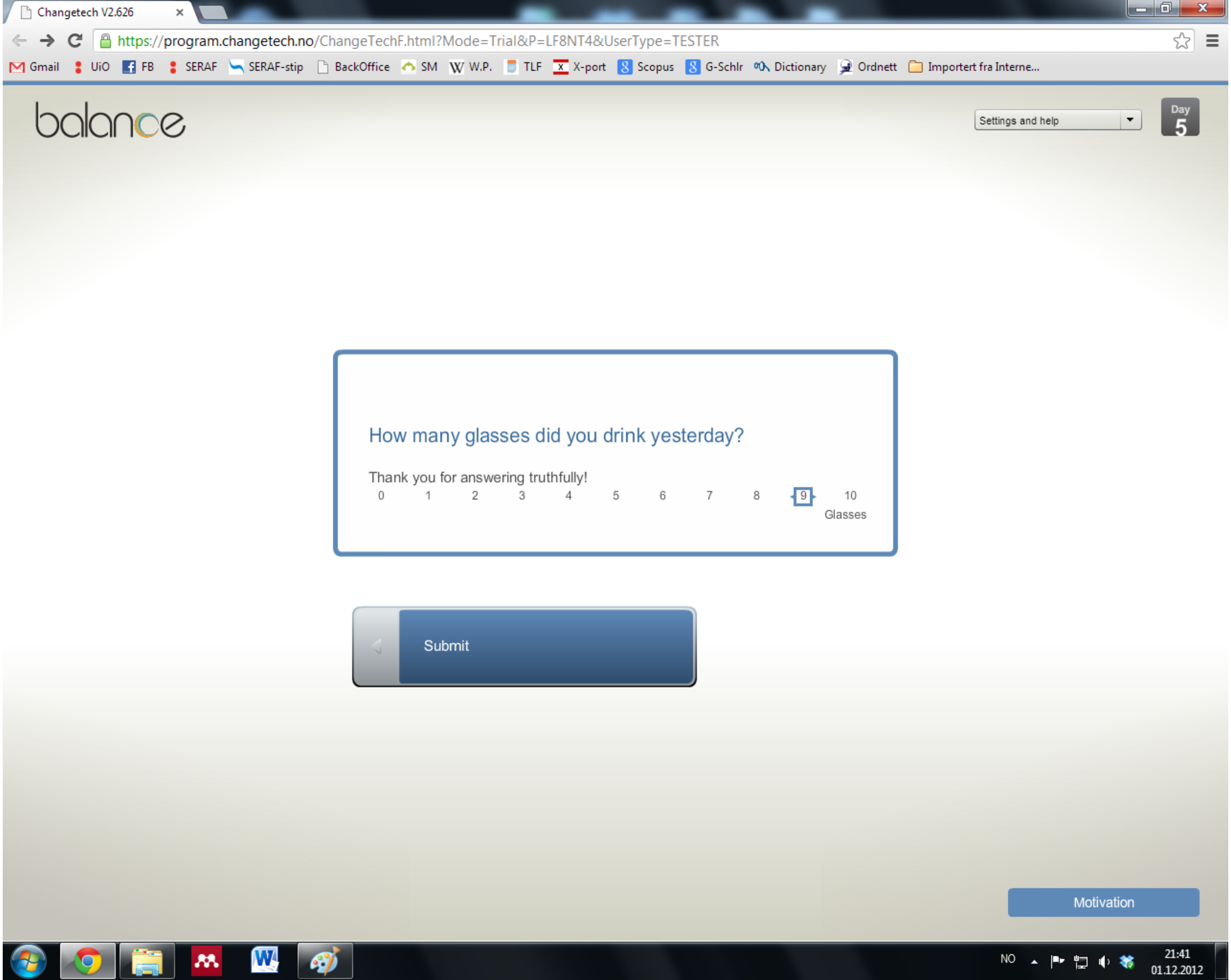
Changetech V2.626
https://program.changetech.no/ChangeTechF.html?Mode=Trial&P=LF8NT4&UserType=TESTER
Gmail UiO FB SERAF SERAF-stip BackOffice SM W.P. TLF X-port Scopus G-Schlr Dictionary Ordnett Importert fra Interne...
balance
Settings and help
Day 5
How many glasses did you drink yesterday?
Thank you for answering truthfully!
0 1 2 3 4 5 6 7 8 9 10
Glasses
Submit
Motivation
NO
21:41
01.12.2012

Changetech V2.626

https://program.changetech.no/ChangeTechF.html?Mode=Trial&P=LF8NT4&UserType=TESTER

Gmail UiO FB SERAF SERAF-stip BackOffice SM W.P. TLF X-port Scopus G-Schlr Dictionary Ordnett Importert fra Interne...

balance

Settings and help

Day 7

Well done!

You drank less than your goal yesterday!

How about today?

Motivation

NO 22:01 01.12.2012

Changetech V2.626

https://program.changetech.no/ChangeTechF.html?Mode=Trial&P=LF8NT4&UserType=TESTER

Gmail UiO FB SERAF SERAF-stip BackOffice SM W.P. TLF X-port Scopus G-Schlr Dictionary Ordnett Importert fra Interne...

balance

Settings and help

Day 5

What happened yesterday?

Was it just a slip-up or do you think you are about to crack?

- (•) It was a slip-up
- ( ) I think I'm close to cracking

Fine. Then let's carry on with today's programme.

Continue

Motivation

NO 21:43 01.12.2012

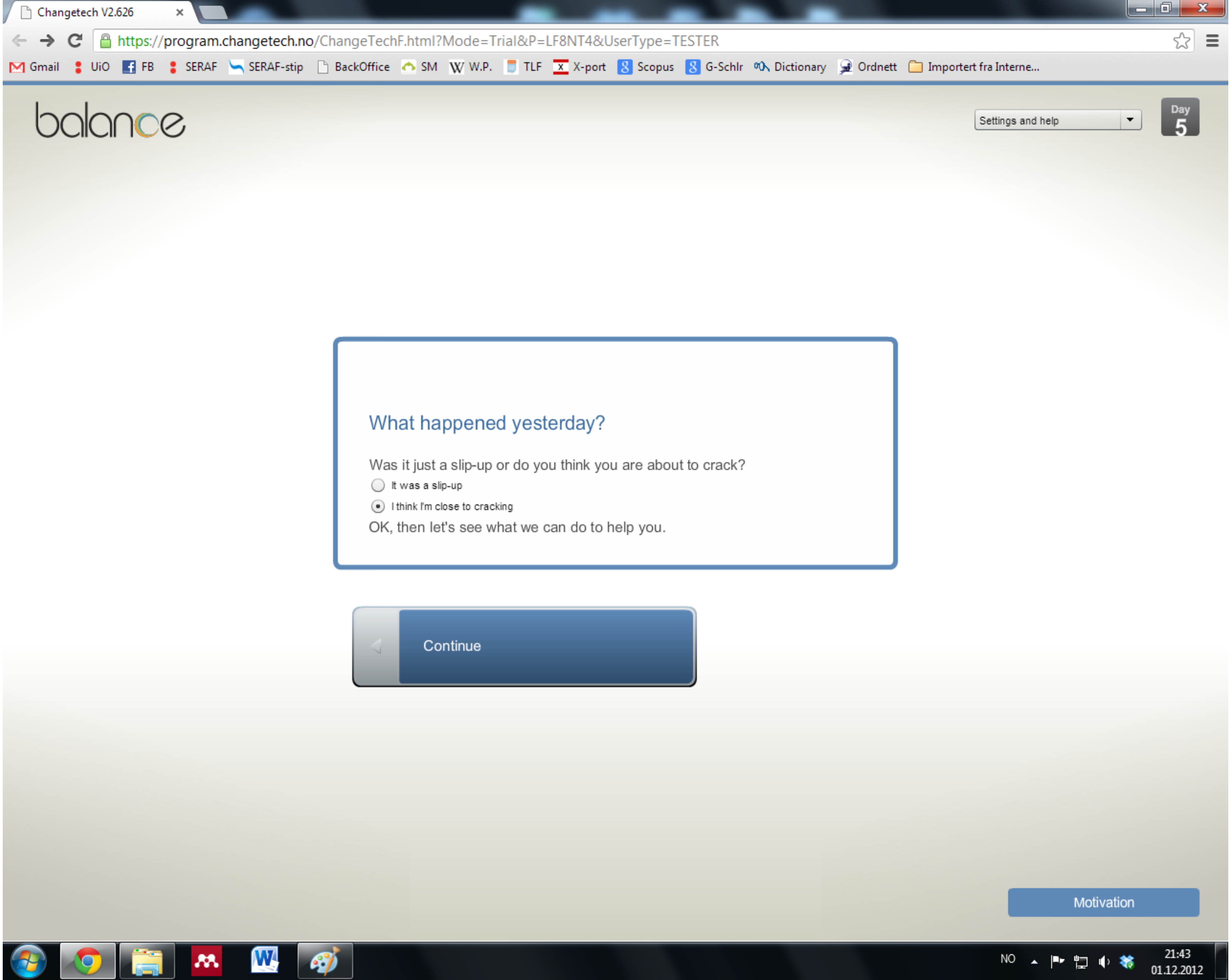
Changetech V2.626
https://program.changetech.no/ChangeTechF.html?Mode=Trial&P=LF8NT4&UserType=TESTER
Gmail
UiO
FB
SERAF
SERAF-stip
BackOffice
SM
W.P.
TLF
X-port
Scopus
G-Schlr
Dictionary
Ordnett
Importert fra Interne...
balance
Settings and help
Day 5
What happened yesterday?
Was it just a slip-up or do you think you are about to crack?
It was a slip-up
I think I'm close to cracking
OK, then let's see what we can do to help you.
Continue
Motivation
NO
21:43
01.12.2012

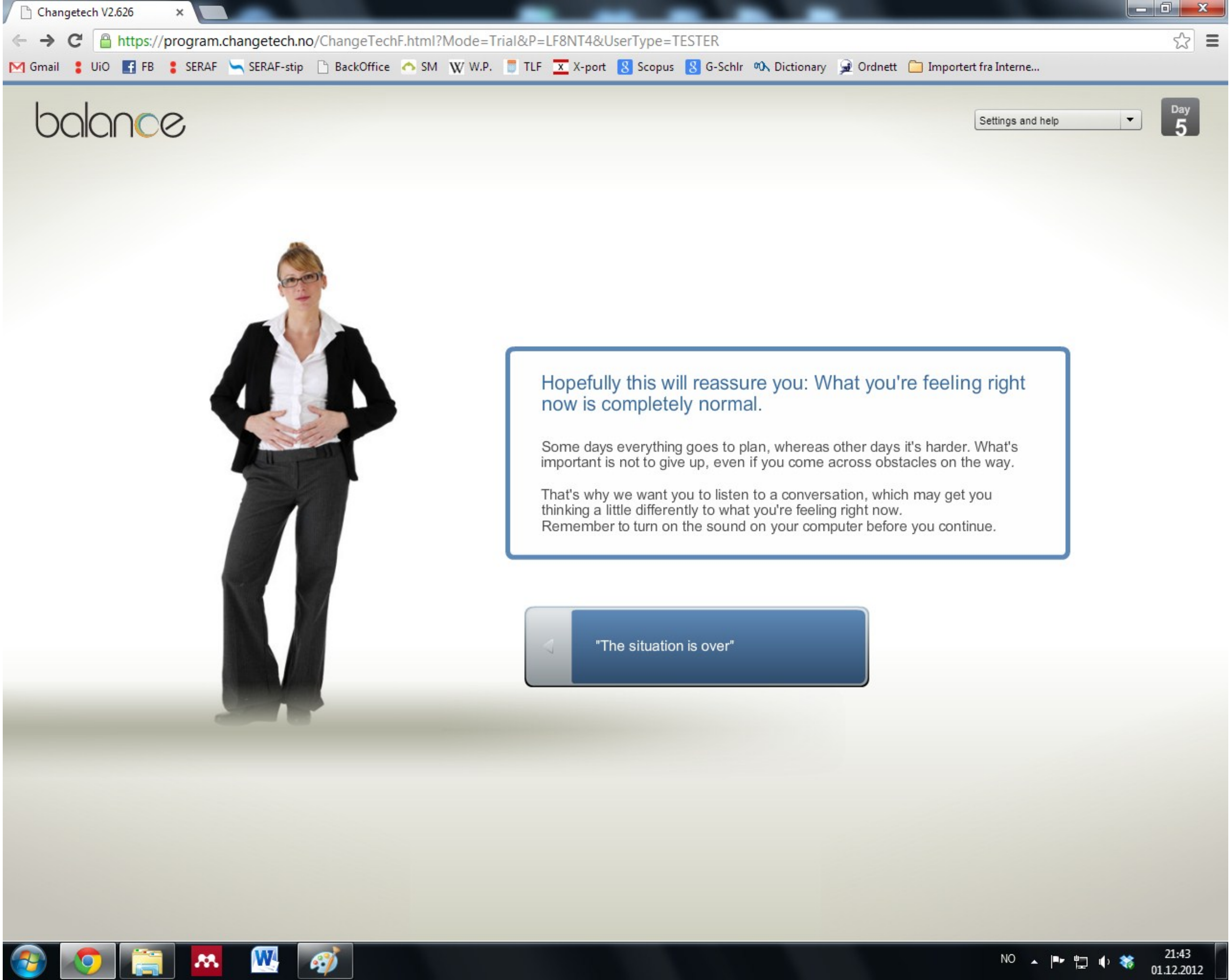
Changetech V2.626
https://program.changetech.no/ChangeTechF.html?Mode=Trial&P=LF8NT4&UserType=TESTER
Gmail UiO FB SERAF SERAF-stip BackOffice SM W.P. TLF X-port Scopus G-Schlr Dictionary Ordnett Importert fra Interne...
balance
Settings and help
Day 5
Hopefully this will reassure you: What you're feeling right now is completely normal.
Some days everything goes to plan, whereas other days it's harder. What's important is not to give up, even if you come across obstacles on the way.
That's why we want you to listen to a conversation, which may get you thinking a little differently to what you're feeling right now.
Remember to turn on the sound on your computer before you continue.
"The situation is over"
NO
21:43
01.12.2012

Changetech V2.626
https://program.changetech.no/ChangeTechF.html?Mode=Trial&P=LF8NT4&UserType=TESTER
Gmail UiO FB SERAF SERAF-stip BackOffice SM W.P. TLF X-port Scopus G-Schlr Dictionary Ordnett Importert fra Interne...

balance

Settings and help

Day 5

Listen to this conversation.

You'll now hear a conversation between someone who started to think about their drinking habits and an expert in the area.

It's a real conversation, but reproduced by two actors.

Click the "Play button". You may have to wait a bit for the sound, depending on how fast your Internet connection is.

Click here when you finish listening

NO 21:44 01.12.2012

- The audio conversation is transcribed verbatim and provided in another appendix

Changetech V2.626

https://program.changetech.no/ChangeTechF.html?Mode=Trial&P=LF8NT4&UserType=TESTER

Gmail UiO FB SERAF SERAF-stip BackOffice SM W.P. TLF X-port Scopus G-Schlr Dictionary Ordnett Importert fra Interne...

balance

Settings and help

Day 5

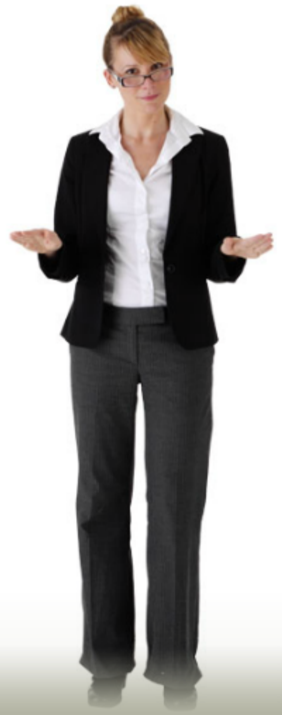

Small setback. One of the things to get over.

What you've just heard is very typical. Someone's experiencing a small setback and thinks: "Oh well, now I've done it" "I might as well begin to smoke again like before". Or "I might as well drink again like before." "I've failed".

No! You haven't.

You must see it as a slip-up, and nothing other than that. And the reason is beside the point.

The one thing you need to do now is tuck away what happened as a useful experience, put your chin up and carry on with today's programme. As if nothing has happened.

OK?

Back to today's Balance

NO 21:44 01.12.2012

Changetech V2.626

https://program.changetech.no/ChangeTechF.html?Mode=Trial&P=LF8NT4&UserType=TESTER

Gmail UiO FB SERAF SERAF-stip BackOffice SM W.P. TLF X-port Scopus G-Schlr Dictionary Ordnett Importert fra Interne...

balance

Settings and help

Day 5

And how do you think you will get on today?

Are you confident that you will achieve your goal for today, which is 8 glasses?

Or are you worried that you will not manage to achieve your goal?

- I'm fairly certain I'll manage
- I'm not sure

Continue

Motivation

NO 21:45 01.12.2012

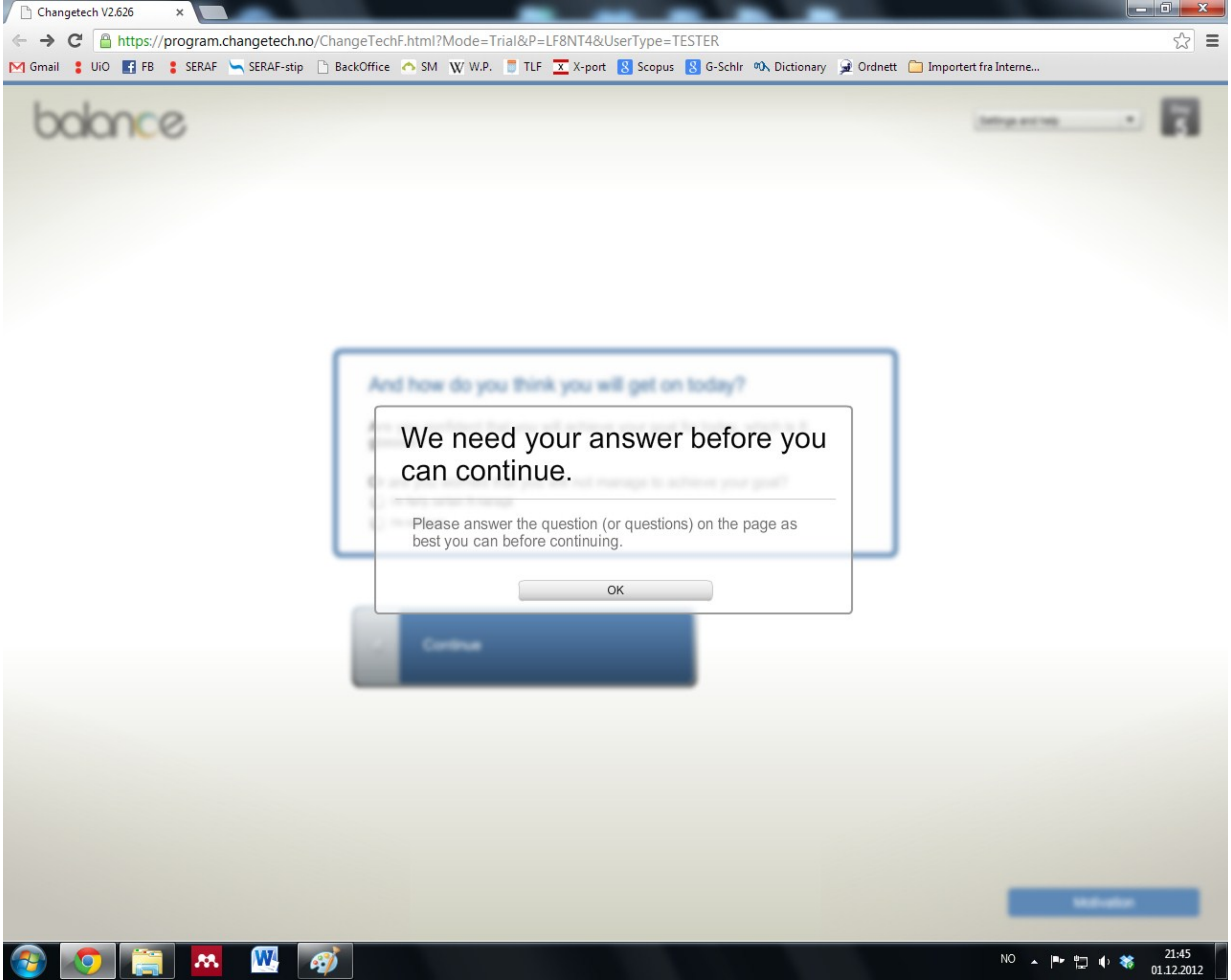
Changetech V2.626
https://program.changetech.no/ChangeTechF.html?Mode=Trial&P=LF8NT4&UserType=TESTER
Gmail
UiO
FB
SERAF
SERAF-stip
BackOffice
SM
W.P.
TLF
X-port
Scopus
G-Schlr
Dictionary
Ordnett
Importert fra Interne...
balance
And how do you think you will get on today?
We need your answer before you can continue.
Please answer the question (or questions) on the page as best you can before continuing.
OK
Continue
NO
21:45
01.12.2012

Changetech V2.626

https://program.changetech.no/ChangeTechF.html?Mode=Trial&P=LF8NT4&UserType=TESTER

Gmail UiO FB SERAF SERAF-stip BackOffice SM W.P. TLF X-port Scopus G-Schlr Dictionary Ordnett Importert fra Interne...

balance

Settings and help

Day 5

And how do you think you will get on today?

Are you confident that you will achieve your goal for today, which is 8 glasses?

Or are you worried that you will not manage to achieve your goal?

- (•) I'm fairly certain I'll manage
- ( ) I'm not sure

Good. Then let's carry on with today's programme.

Continue

Motivation

NO 21:46 01.12.2012

Changetech V2.626
https://program.changetech.no/ChangeTechF.html?Mode=Trial&P=LF8NT4&UserType=TESTER
Gmail UiO FB SERAF SERAF-stip BackOffice SM W.P. TLF X-port Scopus G-Schlr Dictionary Ordnett Importert fra Interne...

balance

Settings and help

Day 5

And how do you think you will get on today?

Are you confident that you will achieve your goal for today, which is 8 glasses?

Or are you worried that you will not manage to achieve your goal?

- ( ) I'm fairly certain I'll manage
- (•) I'm not sure

OK, could you tell us a little bit more?

Continue

Motivation

NO 21:46 01.12.2012

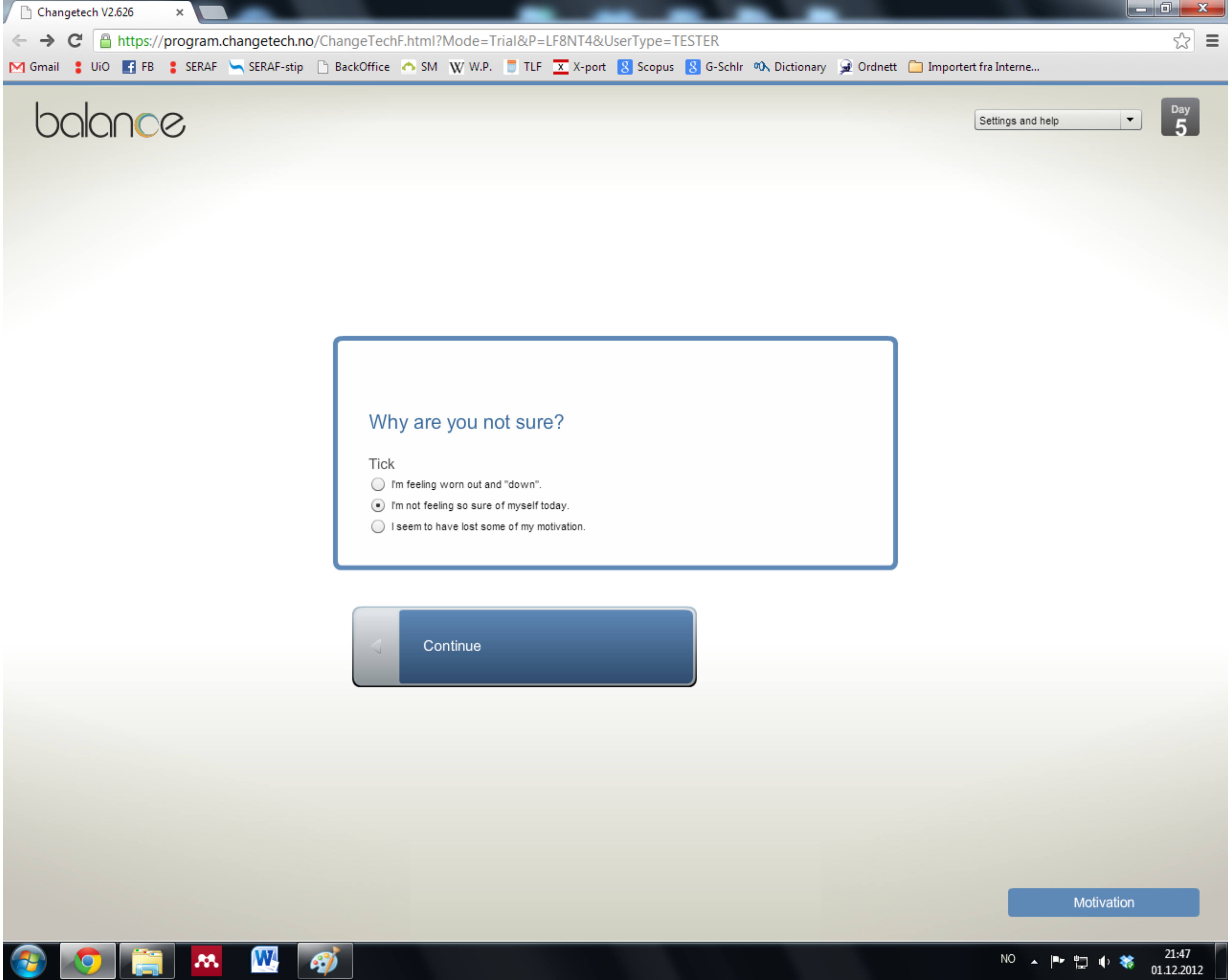
Changetech V2.626
https://program.changetech.no/ChangeTechF.html?Mode=Trial&P=LF8NT4&UserType=TESTER
Gmail UiO FB SERAF SERAF-stip BackOffice SM W.P. TLF X-port Scopus G-Schlr Dictionary Ordnett Importert fra Interne...
balance
Settings and help
Day 5
Why are you not sure?
Tick
I'm feeling worn out and "down".
I'm not feeling so sure of myself today.
I seem to have lost some of my motivation.
Continue
Motivation
NO
21:47
01.12.2012

Changetech V2.626

https://program.changetech.no/ChangeTechF.html?Mode=Trial&P=LF8NT4&UserType=TESTER

Gmail UiO FB SERAF SERAF-stip BackOffice SM W.P. TLF X-port Scopus G-Schlr Dictionary Ordnett Importert fra Interne...

balance

Settings and help

Day 5

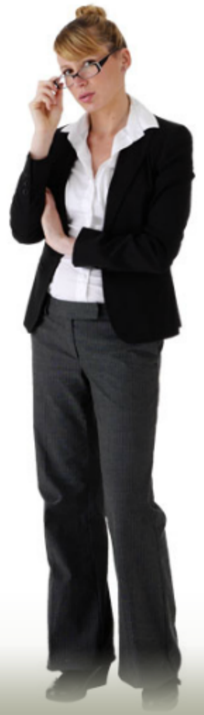

Thank you for telling us!

What you are feeling is completely normal for someone who's trying to change their habits.

When you have a bad day or feel like you've lost courage, you automatically start to wonder whether you will manage. And this is precisely your biggest enemy right now - the idea that you may not succeed.

This is what we'll help you get rid of!

But first, two things

NO 21:47 01.12.2012

Changetech V2.626

https://program.changetech.no/ChangeTechF.html?Mode=Trial&P=LF8NT4&UserType=TESTER

Gmail | UiO | FB | SERAF | SERAF-stip | BackOffice | SM | W.P. | TLF | X-port | Scopus | G-Schlr | Dictionary | Ordnett | Importert fra Interne...

balance

Settings and help

Day 5

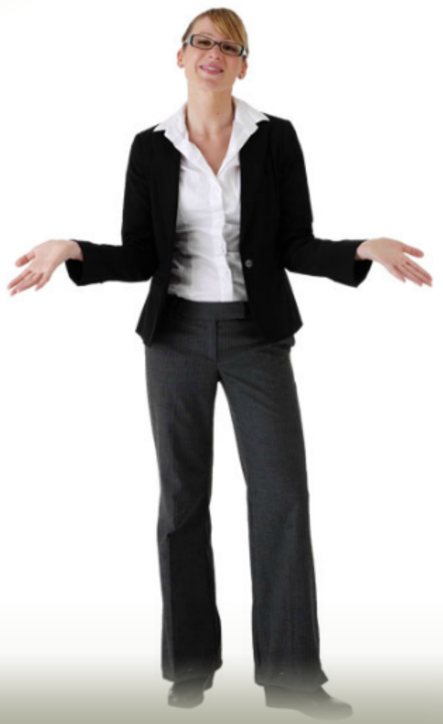

This too will pass!

It will, all by itself. It's just a thought. And thoughts come and go, as you know. But the help you'll get right now will make it disappear more quickly.

The other thing we want to say is that you must believe in the help you get. That's important if you're to get the full effect. Even if what we ask you to do seems childish and naive sometimes.

Everything in Balance is based on recognised psychological principles which have been proven to work. This also applies to the treatment you're getting now.

Here's what you should do

NO 21:47 01.12.2012

balance

Settings and help

Day 5

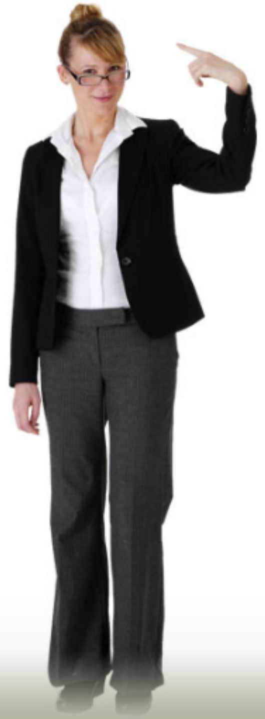

You must talk to yourself!

Very simply a pep talk. You may already know this technique, but it is no less effective.

Say it out loud to yourself:

I want to change my habits.
I want to because I want a good life.
I make the decisions concerning me.
And I've decided to achieve my goals today.

Repeat it several times. Of course, you can also find your own things you want to say. Talk to yourself like a good friend or as if you were your own personal trainer.

Click here when you're finished

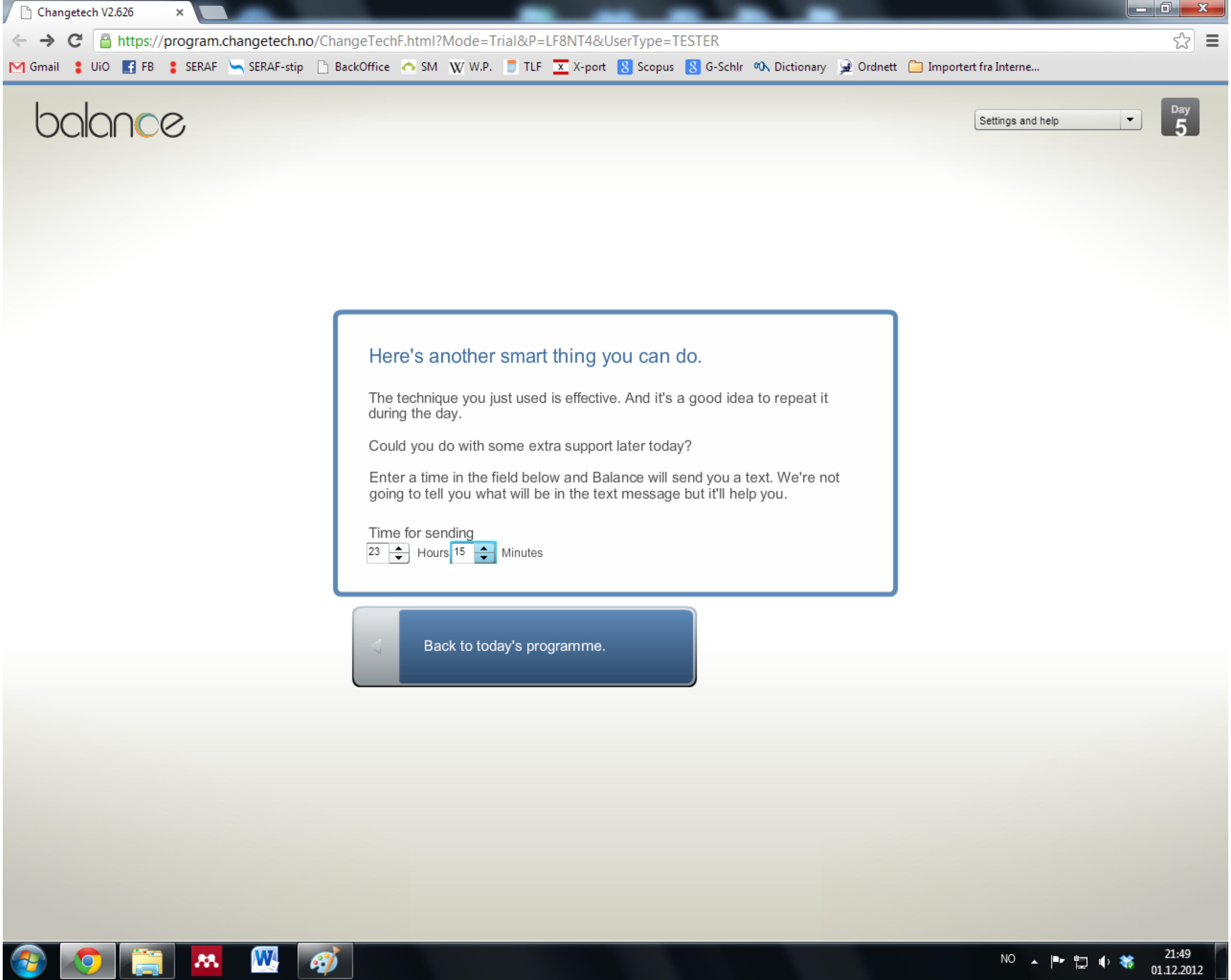
Changetech V2.626
https://program.changetech.no/ChangeTechF.html?Mode=Trial&P=LF8NT4&UserType=TESTER
Gmail UiO FB SERAF SERAF-stip BackOffice SM W.P. TLF X-port Scopus G-Schlr Dictionary Ordnett Importert fra Interne...
balance
Settings and help
Day 5
Here's another smart thing you can do.
The technique you just used is effective. And it's a good idea to repeat it during the day.
Could you do with some extra support later today?
Enter a time in the field below and Balance will send you a text. We're not going to tell you what will be in the text message but it'll help you.
Time for sending
23 Hours 15 Minutes
Back to today's programme.
NO
21:49
01.12.2012

Mobile phone text-message:

Repeat in your head:

"I have the goal because it is good for me. I’m strong enough to manage it. Therefore, I’m going to stick to my goal tonight."

Have a good time!

Self-efficacy

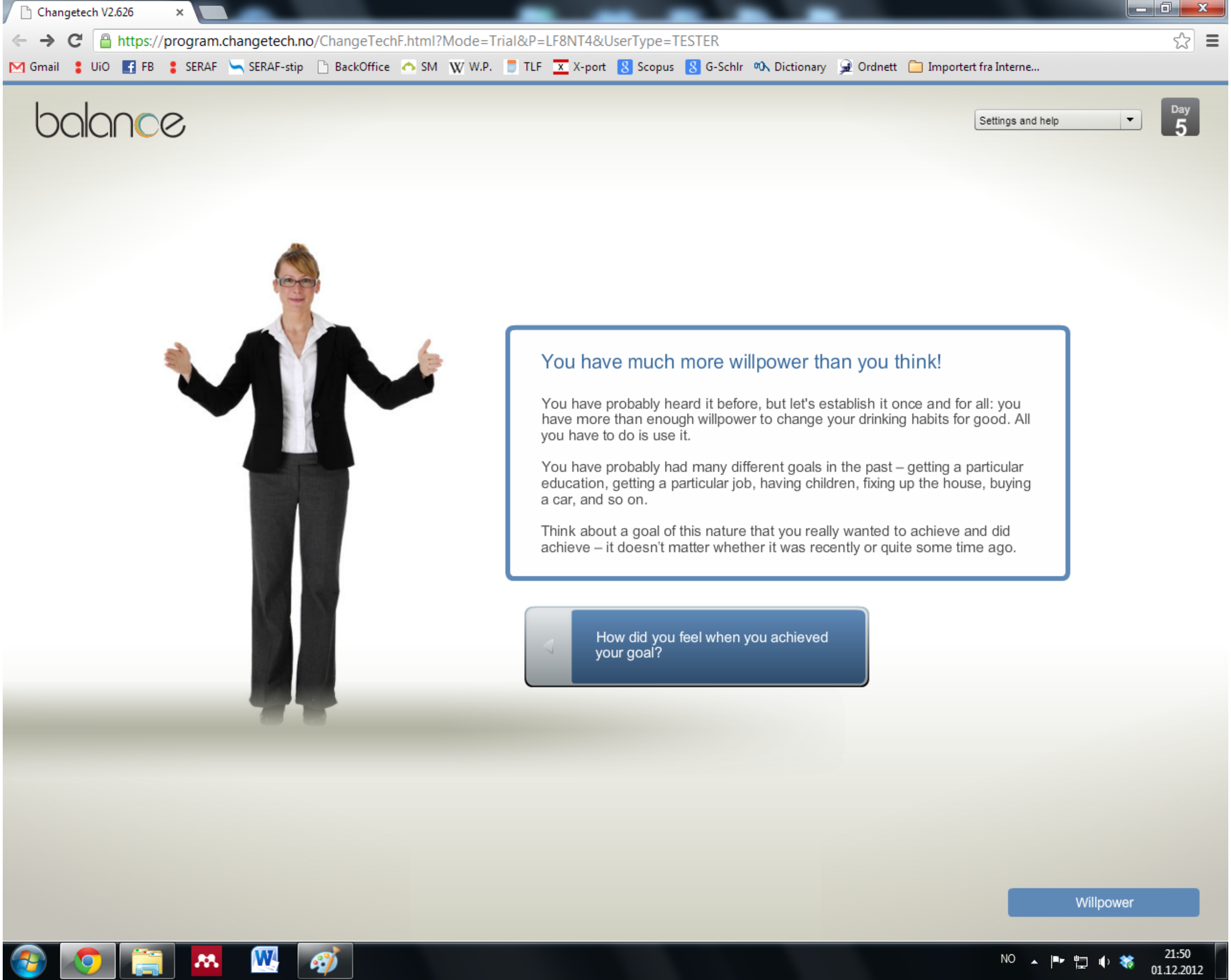
Changetech V2.626
https://program.changetech.no/ChangeTechF.html?Mode=Trial&P=LF8NT4&UserType=TESTER
Gmail
UiO
FB
SERAF
SERAF-stip
BackOffice
SM
W.P.
TLF
X-port
Scopus
G-Schlr
Dictionary
Ordnett
Importert fra Interne...
balance
Settings and help
Day
5
You have much more willpower than you think!
You have probably heard it before, but let's establish it once and for all: you have more than enough willpower to change your drinking habits for good. All you have to do is use it.
You have probably had many different goals in the past – getting a particular education, getting a particular job, having children, fixing up the house, buying a car, and so on.
Think about a goal of this nature that you really wanted to achieve and did achieve – it doesn't matter whether it was recently or quite some time ago.
How did you feel when you achieved your goal?
Willpower
NO
21:50
01.12.2012

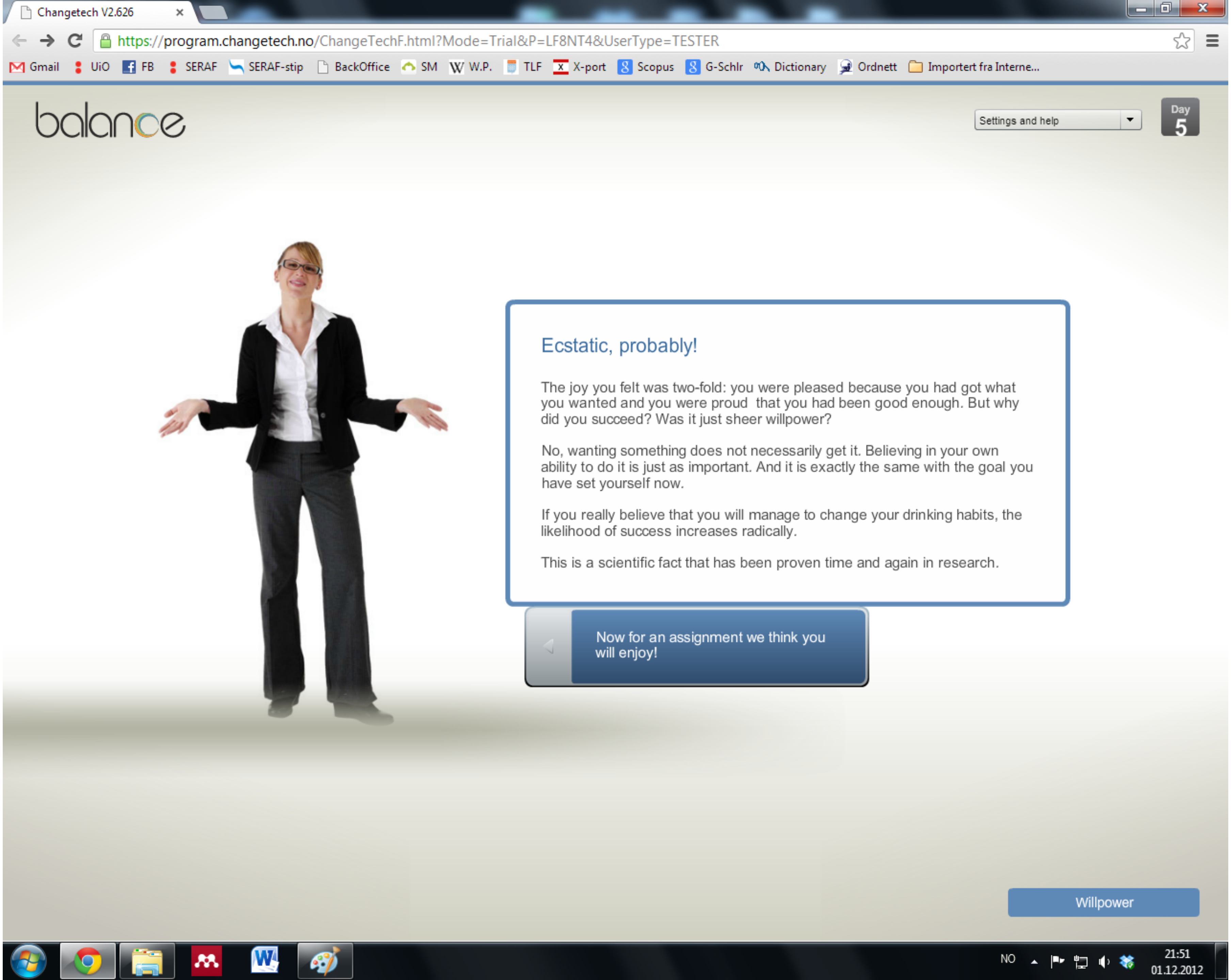
Changetech V2.626
https://program.changetech.no/ChangeTechF.html?Mode=Trial&P=LF8NT4&UserType=TESTER
Gmail UiO FB SERAF SERAF-stip BackOffice SM W.P. TLF X-port Scopus G-Schlr Dictionary Ordnett Importert fra Interne...
balance
Settings and help
Day 5
Ecstatic, probably!
The joy you felt was two-fold: you were pleased because you had got what you wanted and you were proud that you had been good enough. But why did you succeed? Was it just sheer willpower?
No, wanting something does not necessarily get it. Believing in your own ability to do it is just as important. And it is exactly the same with the goal you have set yourself now.
If you really believe that you will manage to change your drinking habits, the likelihood of success increases radically.
This is a scientific fact that has been proven time and again in research.
Now for an assignment we think you will enjoy!
Willpower
NO
21:51
01.12.2012

Changetech V2.626

https://program.changetech.no/ChangeTechF.html?Mode=Trial&P=LF8NT4&UserType=TESTER

Gmail | UiO | FB | SERAF | SERAF-stip | BackOffice | SM | W.P. | TLF | X-port | Scopus | G-Schlr | Dictionary | Ordnett | Importert fra Interne...

balance

Settings and help

Day 5

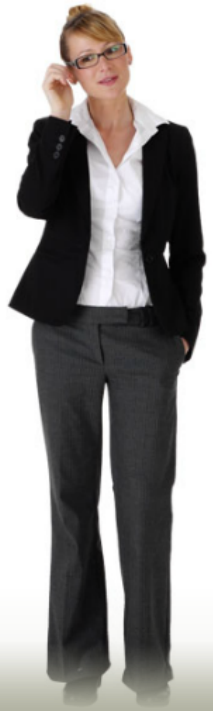

Tell us about your "triumphs"!

You will find that you have achieved many things in your life, if you think about it. Let us explain why we want you to make a list of your accomplishments.

You are on a journey – a journey to change your habits and a number of other things too. In psychology, this is called behaviour change.

An important key to successful behaviour change is believing you can do it.

Your faith in yourself determines the amount of effort you are able to make. By recalling your previous successes, you reinforce that faith.

Think of something you have achieved.

Willpower

NO 21:51 01.12.2012

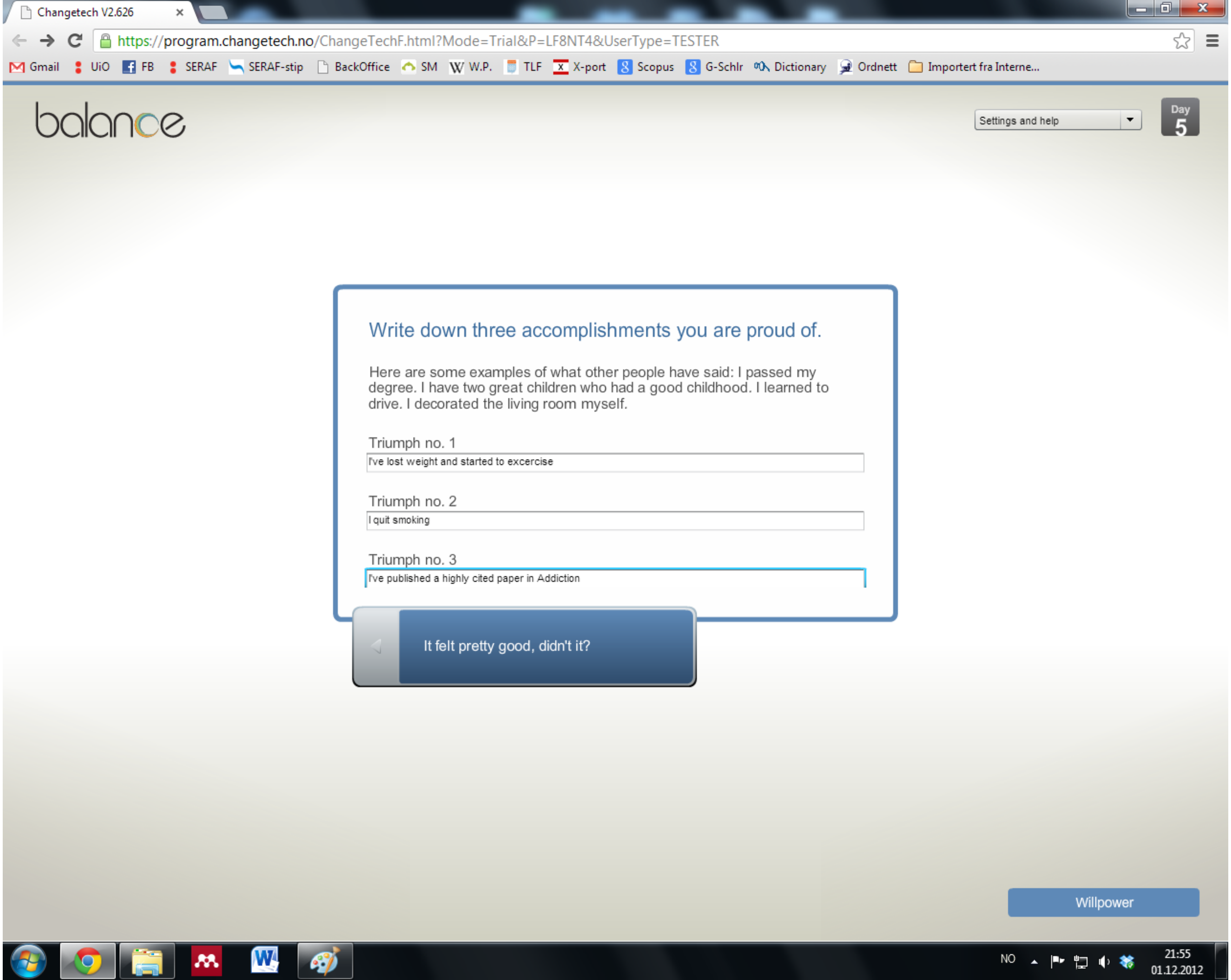
Changetech V2.626
https://program.changetech.no/ChangeTechF.html?Mode=Trial&P=LF8NT4&UserType=TESTER
balance
Settings and help
Day
5
Write down three accomplishments you are proud of.
Here are some examples of what other people have said: I passed my degree. I have two great children who had a good childhood. I learned to drive. I decorated the living room myself.
Triumph no. 1
I've lost weight and started to excercise
Triumph no. 2
I quit smoking
Triumph no. 3
I've published a highly cited paper in Addiction
It felt pretty good, didn't it?
Willpower
21:55
01.12.2012

The setting of and the adjustment of goals and the overview reports

Changetech V2.626

https://program.changetech.no/ChangeTechF.html?Mode=Trial&P=LF8NT4&UserType=TESTER

Gmail | UiO | FB | OBF | SERAF | SERAF-stip | BackOffice | SM | W.P. | TLF | X-port | Scopus | G-Schlr | Dictionary | Ordnett | Importert fra Interne...

balance

Settings and help

Day 1

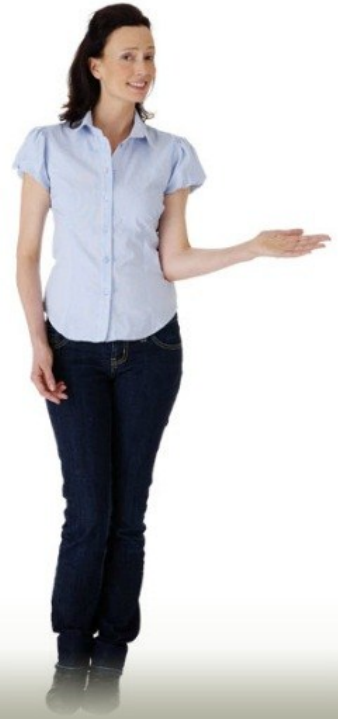

Things you should consider when setting goals.

Do you remember describing how much you drink in a "typical week" when you took the Quick Check? We are going to revisit your typical week now, but this time we want you to enter how many glasses of alcohol you are going to allow yourself during the course of one week.

This is what we call your goals. It is important to set realistic goals that you think you can achieve; not goals that will require a complete lifestyle change. You need to enjoy life too.

You can always modify your goals later on. It is a good idea to start slowly and then cut down even more as you meet your initial goals.

It has taken you a long time to form the habits you have today. Do not try to change them radically overnight.

Now you are ready to enter your goals

Motivation

NO 19:14 01.12.2012

balance

Settings and help

Day 8

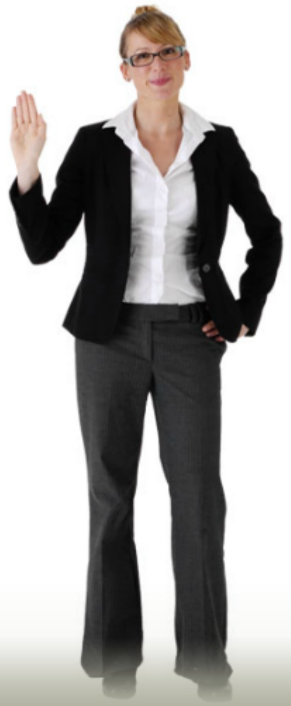

Welcome to week 2 of Balance!

Give yourself a pat on the back for getting through the first week! It will only get easier in the weeks to come.

Today you will be given your first overview report showing how the previous week went. You already know if you have had a slip-up or two. It is not the end of the world if you drank more than you had planned one day.

You must not think:

"I have failed! I might as well go back to my old ways. I can always try to change later on."

A slip-up does NOT mean you have failed

Willpower

balance

Settings and help

Day 8

Here is an overview of your drinking last week.

During the week you drank 9 glasses, whereas your goal was 33 glasses. You have changed the number of glasses you drink from 34 in a typical week to 9 this week.

Monday	0	Your goal was	3
Tuesday	0	Your goal was	3
Wednesday	0	Your goal was	3
Thursday	9	Your goal was	3
Friday	0	Your goal was	8
Saturday	0	Your goal was	10
Sunday	0	Your goal was	3

Continue

Motivation

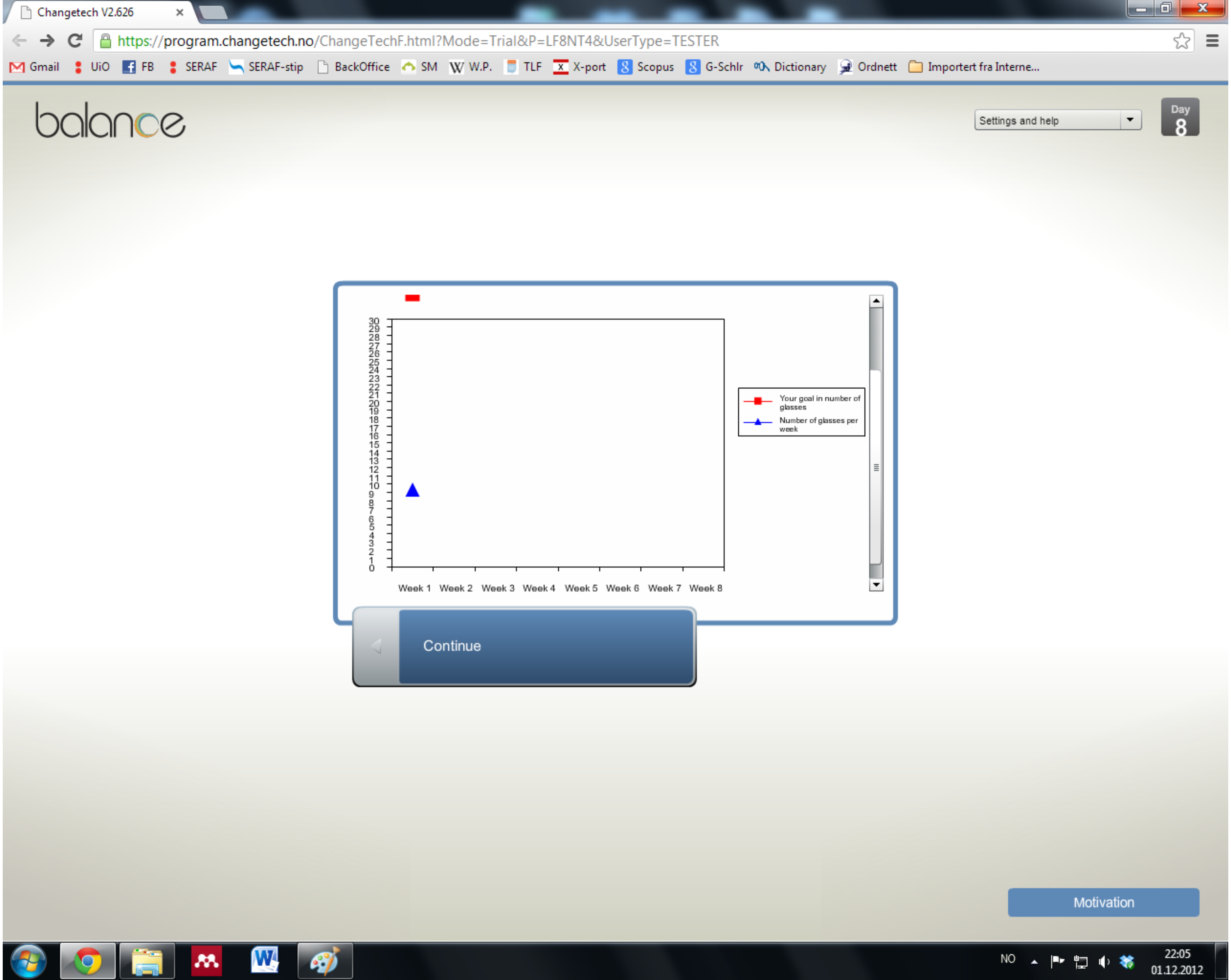
Changetech V2.626
https://program.changetech.no/ChangeTechF.html?Mode=Trial&P=LF8NT4&UserType=TESTER
Gmail
UiO
FB
SERAF
SERAF-stip
BackOffice
SM
W.P.
TLF
X-port
Scopus
G-Schlr
Dictionary
Ordnett
Importert fra Interne...
balance
Settings and help
Day
8
30 29 28 27 26 25 24 23 22 21 20 19 18 17 16 15 14 13 12 11 10 9 8 7 6 5 4 3 2 1 0
Week 1 Week 2 Week 3 Week 4 Week 5 Week 6 Week 7 Week 8
Your goal in number of glasses
Number of glasses per week
Continue
Motivation
NO
22:05
01.12.2012

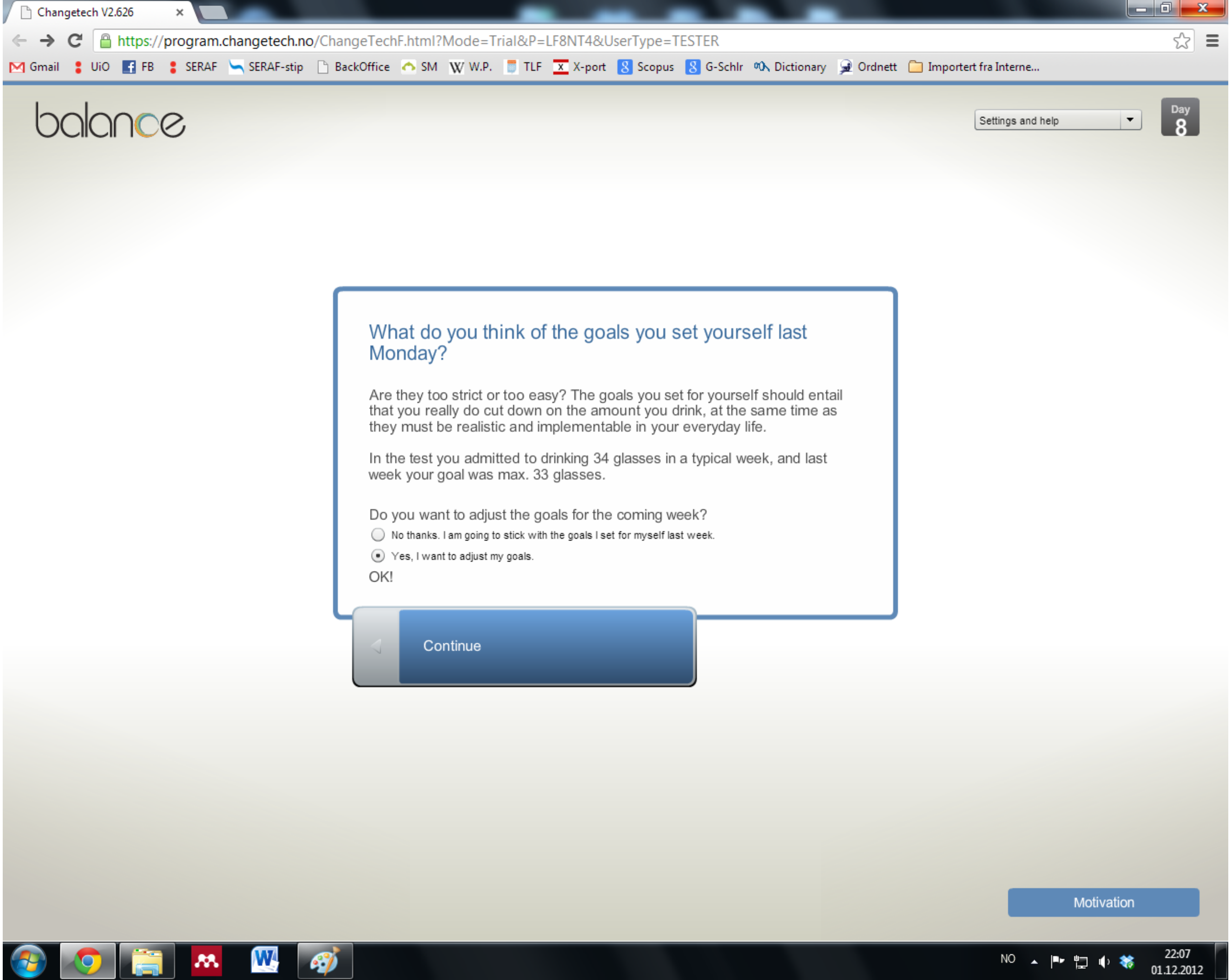
Changetech V2.626
https://program.changetech.no/ChangeTechF.html?Mode=Trial&P=LF8NT4&UserType=TESTER
Gmail UiO FB SERAF SERAF-stip BackOffice SM W.P. TLF X-port Scopus G-Schlr Dictionary Ordnett Importert fra Interne...
balance
Settings and help
Day 8
What do you think of the goals you set yourself last Monday?
Are they too strict or too easy? The goals you set for yourself should entail that you really do cut down on the amount you drink, at the same time as they must be realistic and implementable in your everyday life.
In the test you admitted to drinking 34 glasses in a typical week, and last week your goal was max. 33 glasses.
Do you want to adjust the goals for the coming week?
No thanks. I am going to stick with the goals I set for myself last week.
Yes, I want to adjust my goals.
OK!
Continue
Motivation
NO
22:07
01.12.2012

Changetech V2.626

https://program.changetech.no/ChangeTechF.html?Mode=Trial&P=LF8NT4&UserType=TESTER

Gmail UiO FB SERAF SERAF-stip BackOffice SM W.P. TLF X-port Scopus G-Schlr Dictionary Ordnett Importert fra Interne...

balance

Settings and help

Day 8

Your goal for the week has changed to max. 11 glasses.

Here you can see your new goal broken down into number of glasses for each day of the week:

Monday	0
Tuesday	0
Wednesday	0
Thursday	0
Friday	5
Saturday	5
Sunday	1

Continue

Motivation

NO 22:08 01.12.2012

Pre-programming

Changetech V2.626

https://program.changetech.no/ChangeTechF.html?Mode=Trial&P=LF8NT4&UserType=TESTER

Gmail UiO FB SERAF SERAF-stip BackOffice SM W.P. TLF X-port Scopus G-Schlr Dictionary Ordnett Importert fra Interne...

balance

Settings and help

Day 8

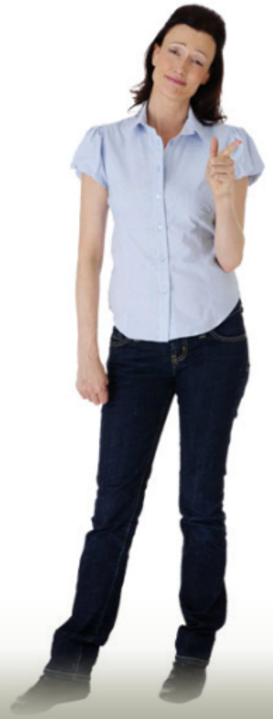

What goes up....can also come down.

Concrete goals, like the ones you have set, are a must when you want to change a habit.

But of course it is not enough just to have goals. You also have to work to achieve them.

Sometimes it seems that everything is going your way and nothing can stop you. At other times you will be tempted to give up. This is simply the way it is.

This week you will learn a new technique called pre-programming. It has proven to be a simple and effective aid in the achievement of goals.

But first a little more about the different phases you will go through

Motivation

NO 22:08 01.12.2012

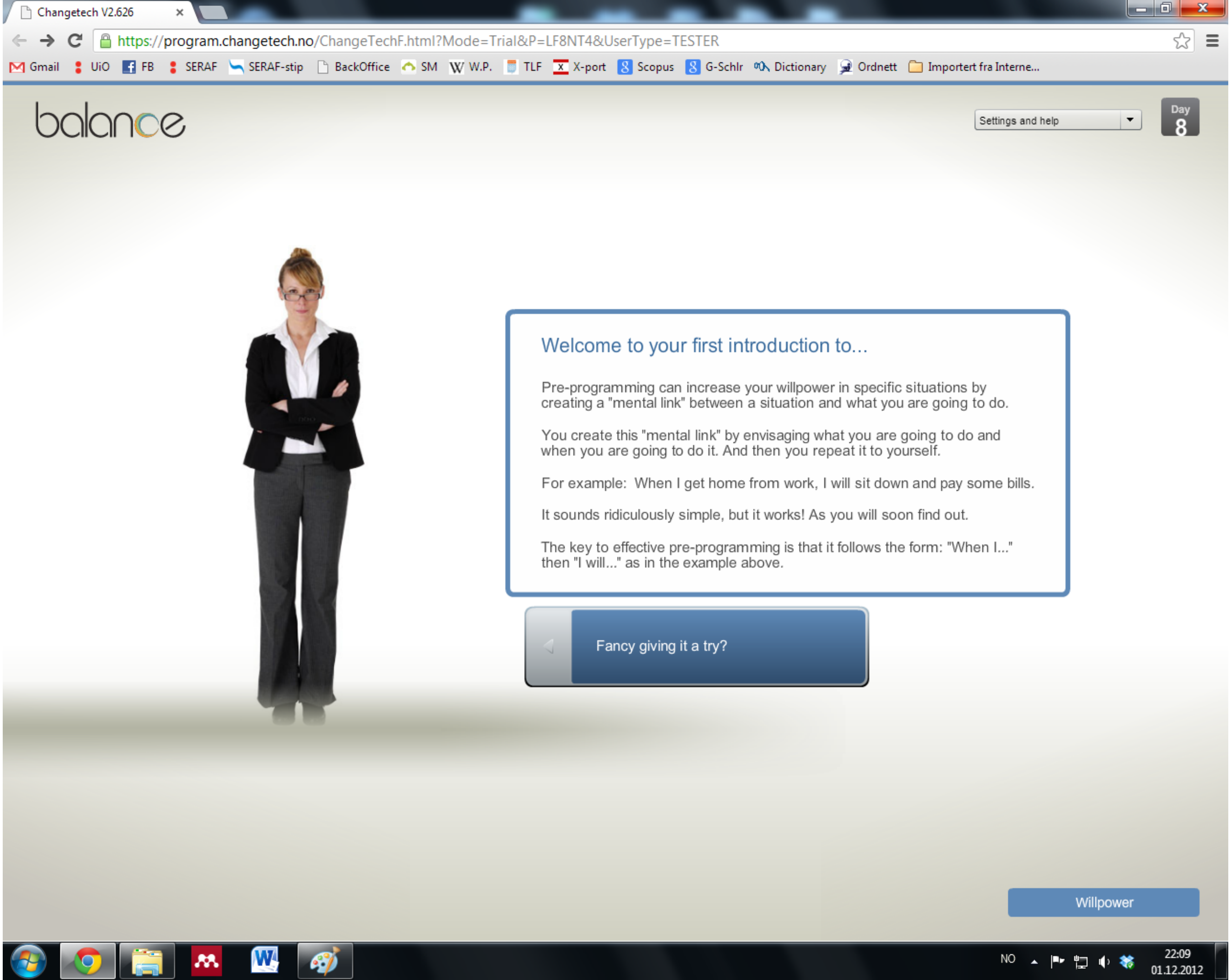
Changetech V2.626
https://program.changetech.no/ChangeTechF.html?Mode=Trial&P=LF8NT4&UserType=TESTER
Gmail UiO FB SERAF SERAF-stip BackOffice SM W.P. TLF X-port Scopus G-Schlr Dictionary Ordnett Importert fra Interne...
balance
Settings and help
Day 8
Welcome to your first introduction to...
Pre-programming can increase your willpower in specific situations by creating a "mental link" between a situation and what you are going to do.
You create this "mental link" by envisaging what you are going to do and when you are going to do it. And then you repeat it to yourself.
For example: When I get home from work, I will sit down and pay some bills.
It sounds ridiculously simple, but it works! As you will soon find out.
The key to effective pre-programming is that it follows the form: "When I..." then "I will..." as in the example above.
Fancy giving it a try?
Willpower
NO
22:09
01.12.2012

Changetech V2.626

https://program.changetech.no/ChangeTechF.html?Mode=Trial&P=LF8NT4&UserType=TESTER

Gmail UiO FB SERAF SERAF-stip BackOffice SM W.P. TLF X-port Scopus G-Schlr Dictionary Ordnett Importert fra Interne...

balance

Settings and help

Day 8

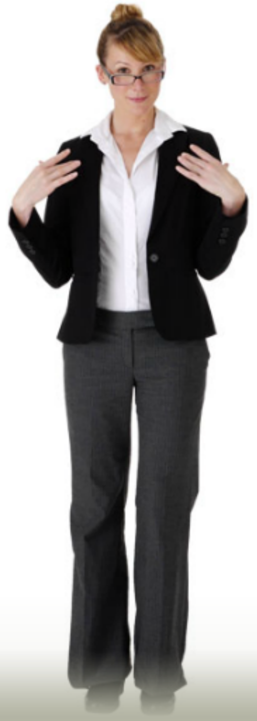

Guess what we think you should pre-program first!

Something you might find easy to forget or avoid ... May we make a suggestion?

We know that if you participate actively in Balance every day and do what we ask, you will succeed in changing your alcohol habits.

So why not try to pre-program your intention to follow Balance every day? You are free to try something else, of course, but why not kill two birds with one stone...?

Here's what you need to do

Willpower

NO 22:09 01.12.2012

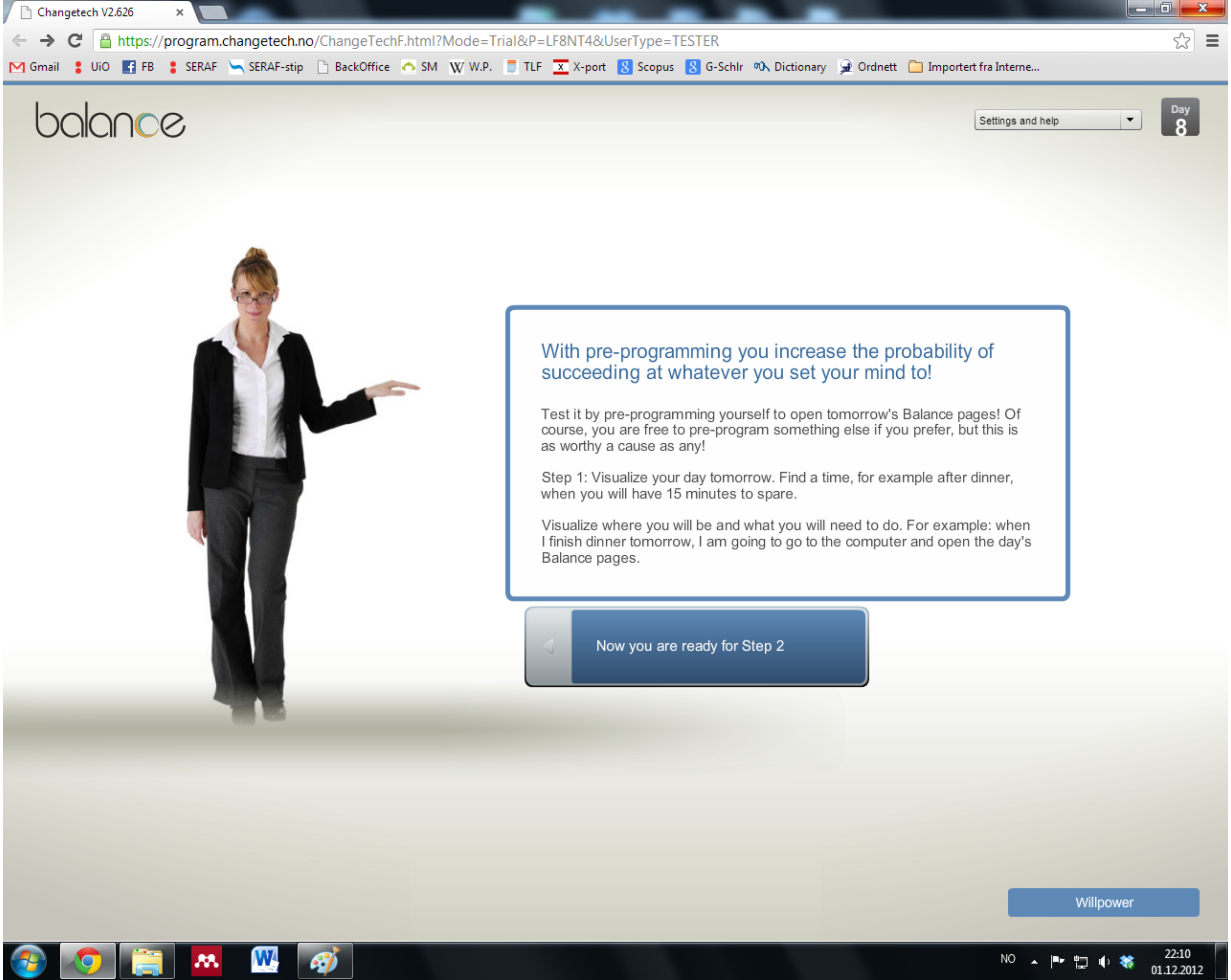
Changetech V2.626
https://program.changetech.no/ChangeTechF.html?Mode=Trial&P=LF8NT4&UserType=TESTER
Gmail UiO FB SERAF SERAF-stip BackOffice SM W.P. TLF X-port Scopus G-Schlr Dictionary Ordnett Importert fra Interne...
balance
Settings and help
Day 8
With pre-programming you increase the probability of succeeding at whatever you set your mind to!
Test it by pre-programming yourself to open tomorrow's Balance pages! Of course, you are free to pre-program something else if you prefer, but this is as worthy a cause as any!
Step 1: Visualize your day tomorrow. Find a time, for example after dinner, when you will have 15 minutes to spare.
Visualize where you will be and what you will need to do. For example: when I finish dinner tomorrow, I am going to go to the computer and open the day's Balance pages.
Now you are ready for Step 2
Willpower
NO
22:10
01.12.2012

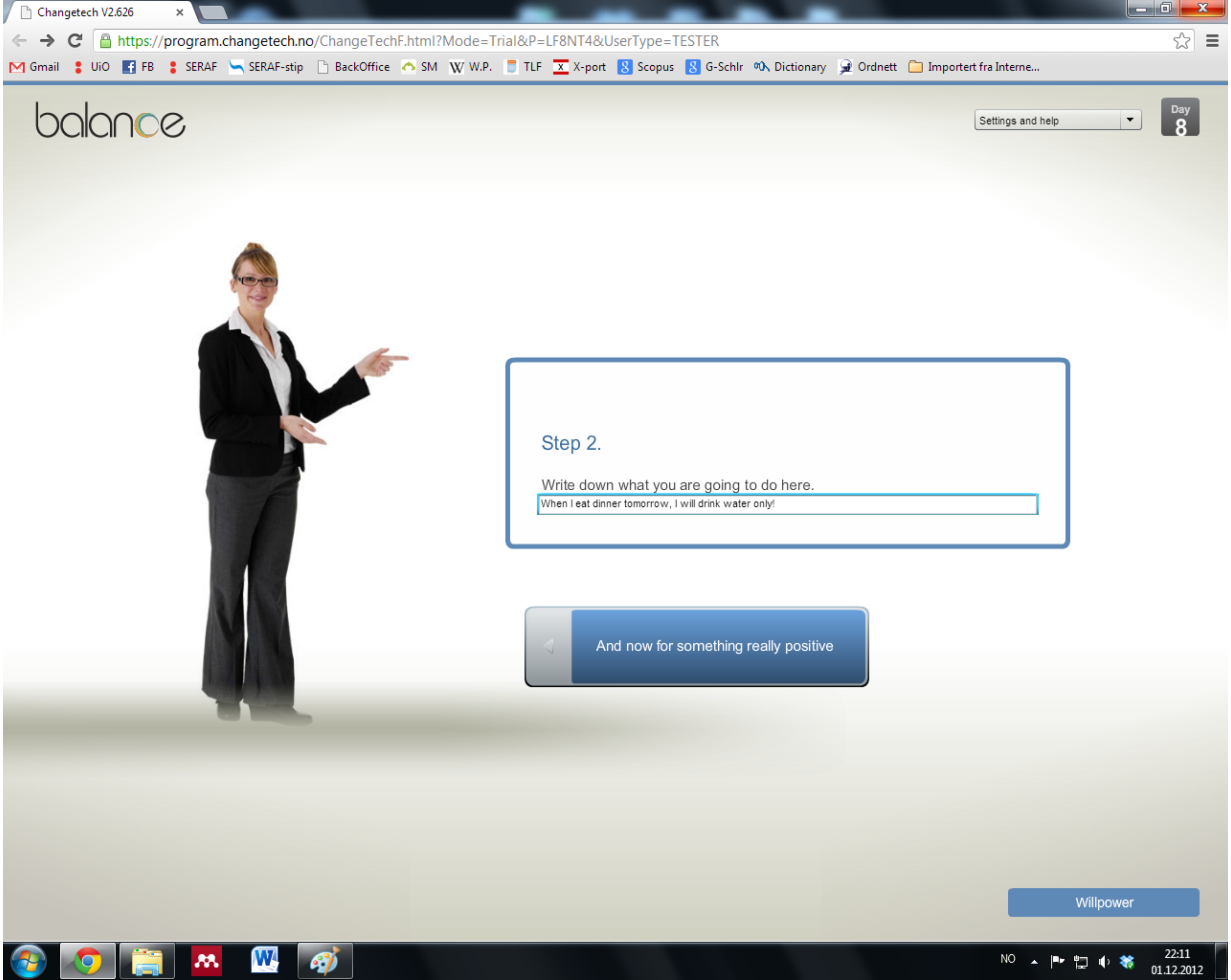
Changetech V2.626
https://program.changetech.no/ChangeTechF.html?Mode=Trial&P=LF8NT4&UserType=TESTER
Gmail UiO FB SERAF SERAF-stip BackOffice SM W.P. TLF X-port Scopus G-Schlr Dictionary Ordnett Importert fra Interne...
balance
Settings and help
Day 8
Step 2.
Write down what you are going to do here.
When I eat dinner tomorrow, I will drink water only!
And now for something really positive
Willpower
NO
22:11
01.12.2012

Good things

A positive psychology exercise

Changetech V2.626

https://program.changetech.no/ChangeTechF.html?Mode=Trial&P=LF8NT4&UserType=TESTER

Gmail UiO FB SERAF SERAF-stip BackOffice SM W.P. TLF X-port Scopus G-Schlr Dictionary Ordnett Importert fra Interne...

balance

Settings and help

Day 8

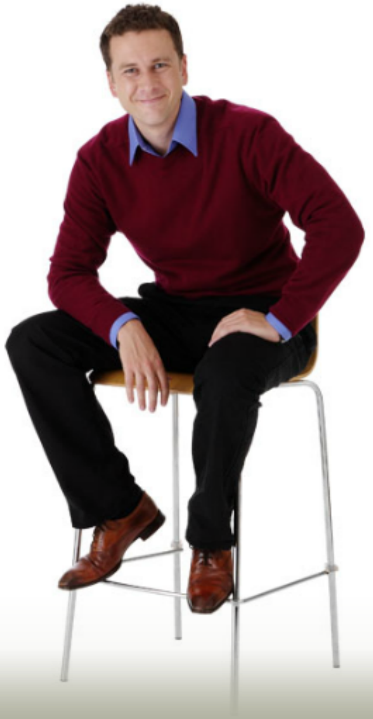

What has been particularly good about today?

Or yesterday, if you are reading this early in the day.

Good things happen every day – some major, some minor – which are easy to overlook and forget. We tend to remember the more negative things, like problems we do not really know how to resolve.

However, recent research shows clearly that our mood improves and we feel better if we manage to hold on to the positive experiences as well.

This is what the new branch of psychology called positive psychology is all about. By thinking about what has gone well today, you can actually control your mood.

Just look at this!

Mood

NO 22:11 01.12.2012

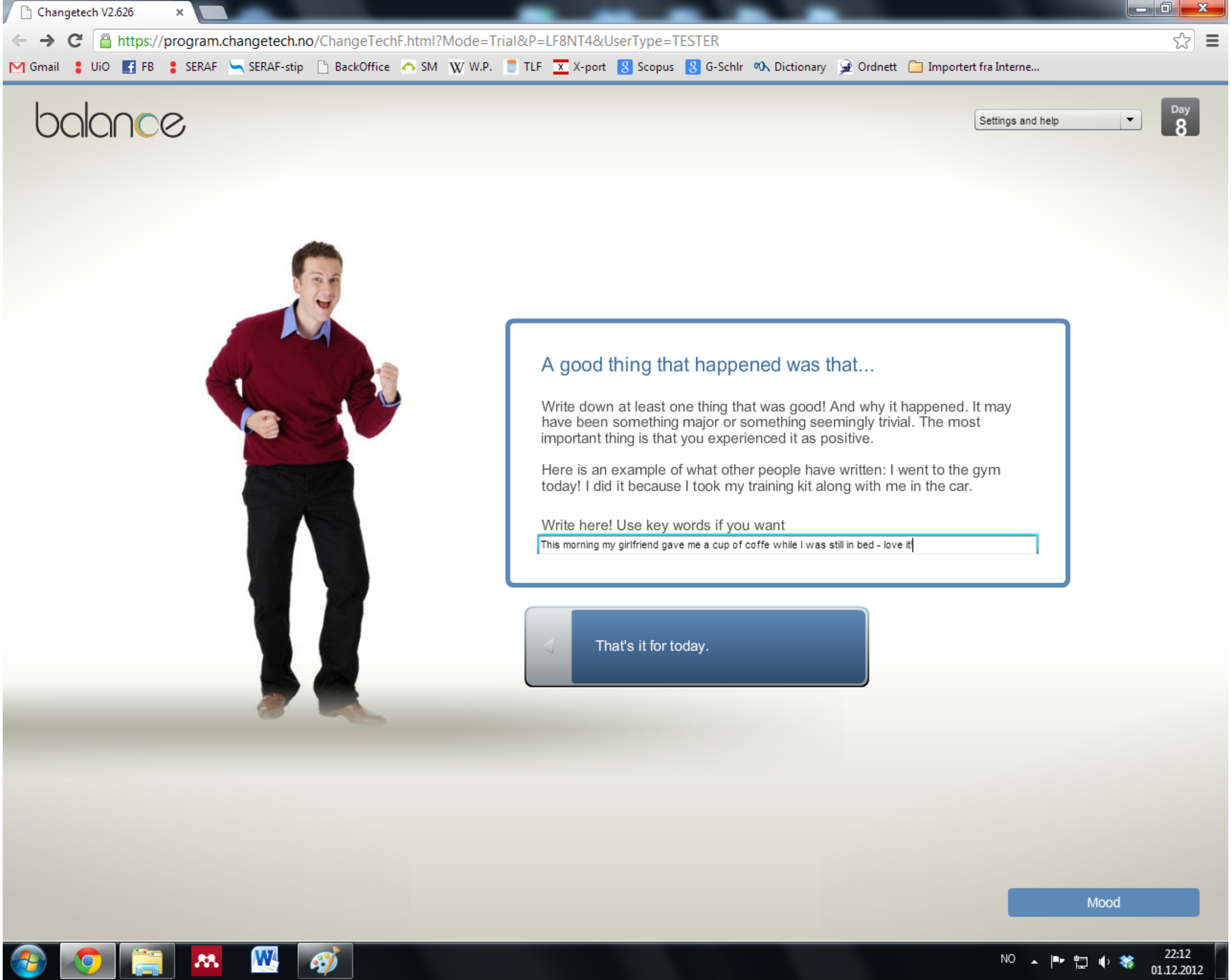
Changetech V2.626
https://program.changetech.no/ChangeTechF.html?Mode=Trial&P=LF8NT4&UserType=TESTER
Gmail UiO FB SERAF SERAF-stip BackOffice SM W.P. TLF X-port Scopus G-Schlr Dictionary Ordnett Importert fra Interne...
balance
Settings and help
Day 8
A good thing that happened was that...
Write down at least one thing that was good! And why it happened. It may have been something major or something seemingly trivial. The most important thing is that you experienced it as positive.
Here is an example of what other people have written: I went to the gym today! I did it because I took my training kit along with me in the car.
Write here! Use key words if you want
This morning my girlfriend gave me a cup of coffe while I was still in bed - love it!
That's it for today.
Mood
NO
22:12
01.12.2012

Changetech V2.626

https://program.changetech.no/ChangeTechF.html?Mode=Trial&P=LF8NT4&UserType=TESTER

Gmail UiO FB SERAF SERAF-stip BackOffice SM W.P. TLF X-port Scopus G-Schlr Dictionary Ordnett Importert fra Interne...

balance

Settings and help

Day 8

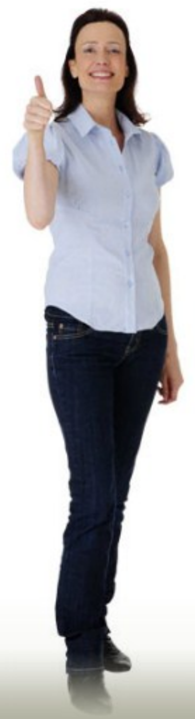

Enjoy the rest of the day ... and the evening!

We look forward to seeing you tomorrow, when we will look at how alcohol affects your body and the two different forms of intoxication.

One of them is pleasant, the other less so.

See you tomorrow!

Exit

Motivation

NO 22:13 01.12.2012

Needs oriented dialogue

Runs over several weeks

Changetech V2.626

https://program.changetech.no/ChangeTechF.html?Mode=Trial&P=LF8NT4&UserType=TESTER

Gmail | UiO | FB | SERAF | SERAF-stip | BackOffice | SM | W.P. | TLF | X-port | Scopus | G-Schlr | Dictionary | Ordnett | Importert fra Interne...

balance

Settings and help

Day 2

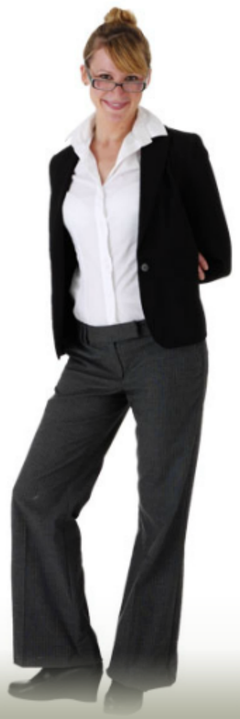

Specially for you.

Yesterday, we asked you to choose between two different options, and you chose the "Needs-oriented dialogue" (NOD) track.

Every Tuesday you will receive some content that has been specially adapted for you, here in the Willpower Room. It will deal with how you can achieve what you want without conflict. NOD is based on recent psychological research linked to how we perceive things, what we feel and what we say.

The overall conclusion is that it is easier to achieve what you want through cooperation rather than through competition and conflict.

See what NOD can do for you

Willpower

NO 21:28 01.12.2012

Changetech V2.626
https://program.changetech.no/ChangeTechF.html?Mode=Trial&P=LF8NT4&UserType=TESTER
Gmail UiO FB SERAF SERAF-stip BackOffice SM W.P. TLF X-port Scopus G-Schlr Dictionary Ordnett Importert fra Interne...

balance

Settings and help

Day 2

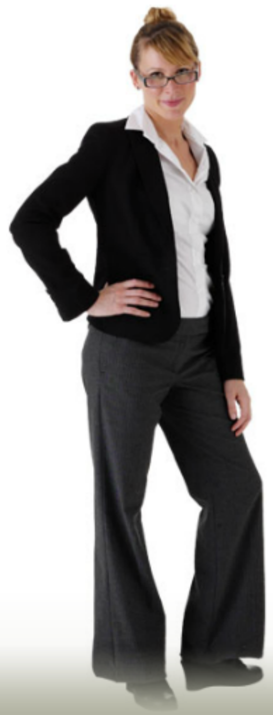

Consider the following conversation between a psychologist and Anders.

Psychologist: You seem to have tried lots of different things to alleviate your back pain?

Anders: Yes, I've tried just about everything, but to no avail. I've just about had enough.

Psychologist: I see. Well, it seems like you've done your best and tried just about everything. But what if there isn't anything to stop the pain?

Anders: What do you mean? Then there's nothing to be done.

Psychologist: I think we need to look into this a bit more deeply. The solutions you have tried don't work. Perhaps these solutions are actually part of the problem?

What do you think the psychologist is trying to say?

Willpower

NO 21:30 01.12.2012

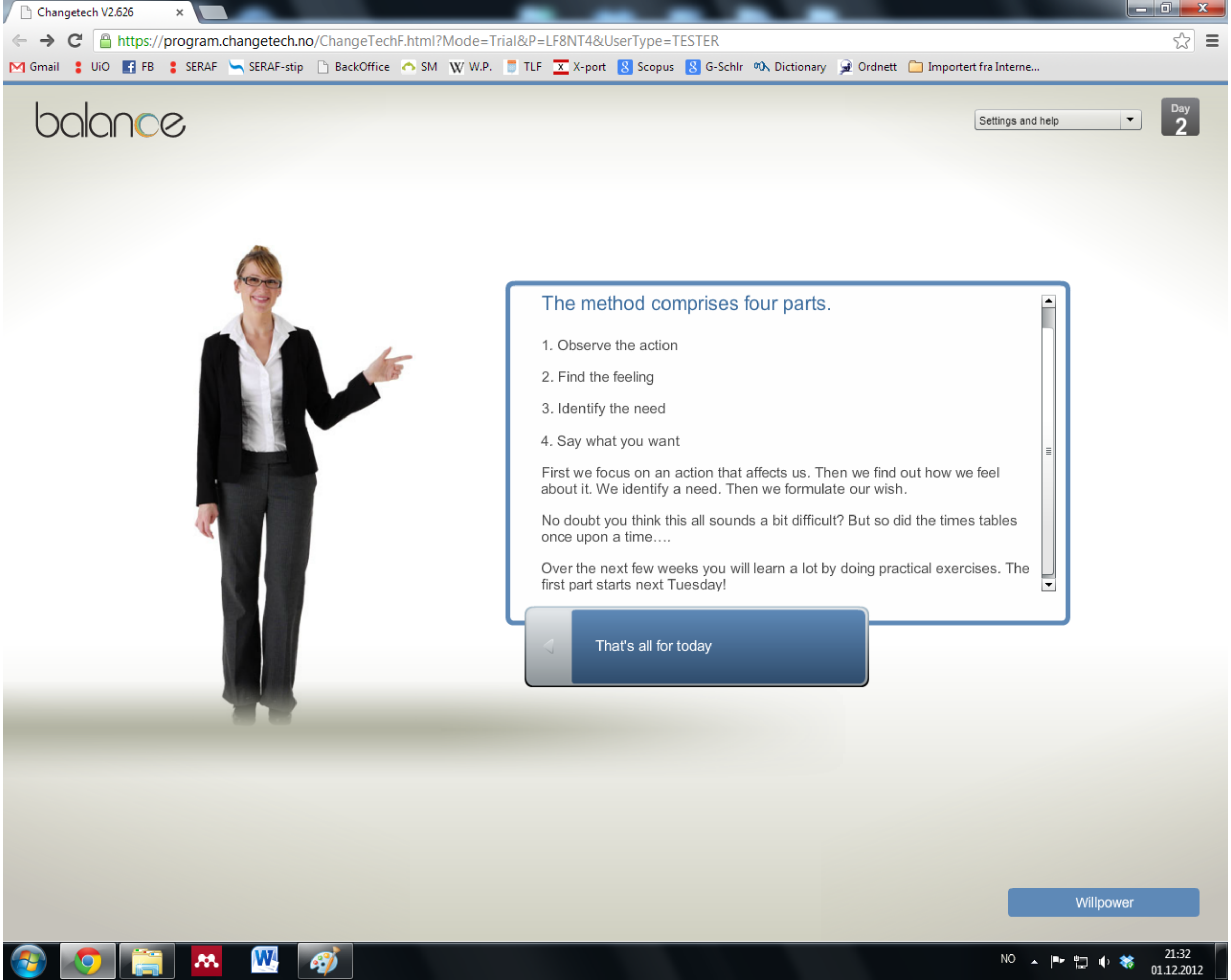
Changetech V2.626
https://program.changetech.no/ChangeTechF.html?Mode=Trial&P=LF8NT4&UserType=TESTER
Gmail UiO FB SERAF SERAF-stip BackOffice SM W.P. TLF X-port Scopus G-Schlr Dictionary Ordnett Importert fra Interne...
balance
Settings and help
Day 2
The method comprises four parts.
1. Observe the action
2. Find the feeling
3. Identify the need
4. Say what you want
First we focus on an action that affects us. Then we find out how we feel about it. We identify a need. Then we formulate our wish.
No doubt you think this all sounds a bit difficult? But so did the times tables once upon a time….
Over the next few weeks you will learn a lot by doing practical exercises. The first part starts next Tuesday!
That's all for today
Willpower
NO
21:32
01.12.2012

Changetech V2.626

https://program.changetech.no/ChangeTechF.html?Mode=Trial&P=LF8NT4&UserType=TESTER

Gmail UiO FB SERAF SERAF-stip BackOffice SM W.P. TLF X-port Scopus G-Schlr Dictionary Ordnett Importert fra Interne...

balance

Settings and help

Day 9

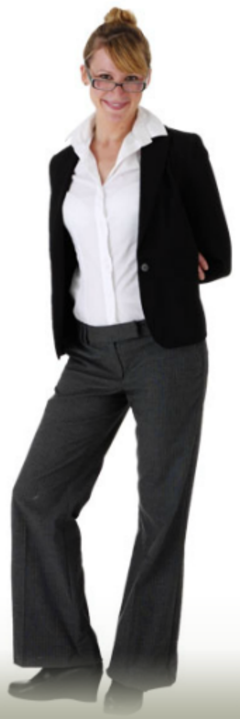

Last week, we introduced "Needs-oriented dialogue" – a communication technique that makes it easier for you to achieve what you want when you talk to others.

As you probably remember, needs-oriented dialogue consists of four elements:

Observe the action.
Identify the feeling.
Identify the need.
State what you want.

Today we are going to look at the first stage: differentiating between an action and your assessment of or feelings about the action.

Needs-oriented dialogue can sound a little abstract to begin with. However, the practical examples below should serve to make it more comprehensible.

It is so easy to criticize

Willpower

NO 22:14 01.12.2012

Changetech V2.626
https://program.changetech.no/ChangeTechF.html?Mode=Trial&P=LF8NT4&UserType=TESTER
Gmail UiO FB SERAF SERAF-stip BackOffice SM W.P. TLF X-port Scopus G-Schlr Dictionary Ordnett Importert fra Interne...

balance

Settings and help

Day 9

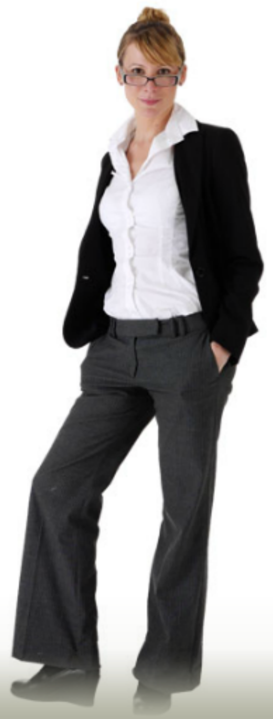

The first step of needs-oriented dialogue entails looking at something that has happened or is happening without making any judgement about it.

Here are a few examples:

Observation: Paul did not start working on the presentation until Monday evening.

Judgement: Paul always leaves things until the very last minute.

Observation: The government has not followed up on its promise to provide day care for all pre-school children.

Judgement: Politicians never keep their promises.

When we combine what we observe with our judgement or feelings about it, other people perceive it as criticism and get defensive, making it almost impossible for us to achieve what we want.

Here is a little exercise

Willpower

NO 22:15 01.12.2012

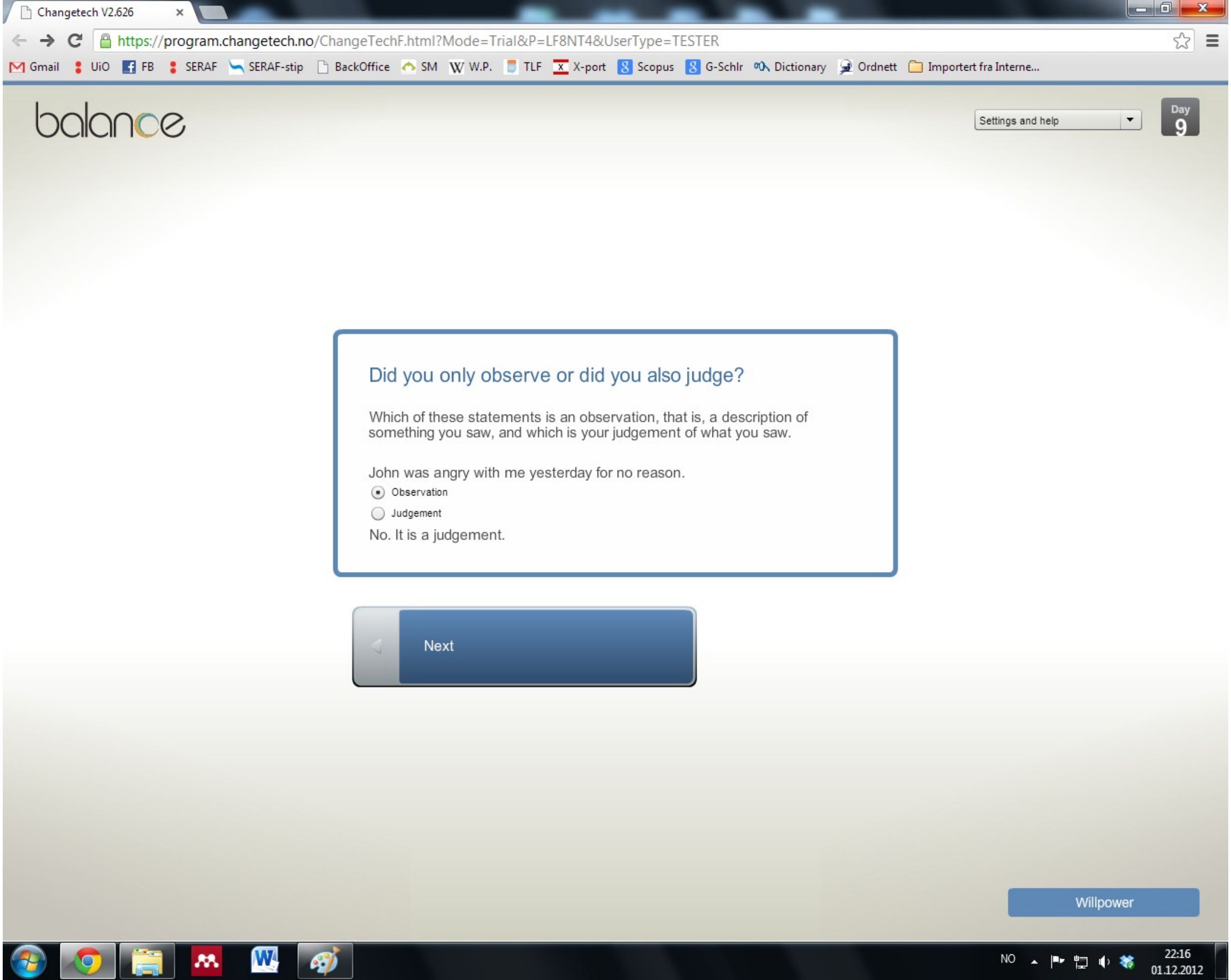
Changetech V2.626
https://program.changetech.no/ChangeTechF.html?Mode=Trial&P=LF8NT4&UserType=TESTER
Gmail UiO FB SERAF SERAF-stip BackOffice SM W.P. TLF X-port Scopus G-Schlr Dictionary Ordnett Importert fra Interne...
balance
Settings and help
Day 9
Did you only observe or did you also judge?
Which of these statements is an observation, that is, a description of something you saw, and which is your judgement of what you saw.
John was angry with me yesterday for no reason.
Observation
Judgement
No. It is a judgement.
Next
Willpower
NO
22:16
01.12.2012

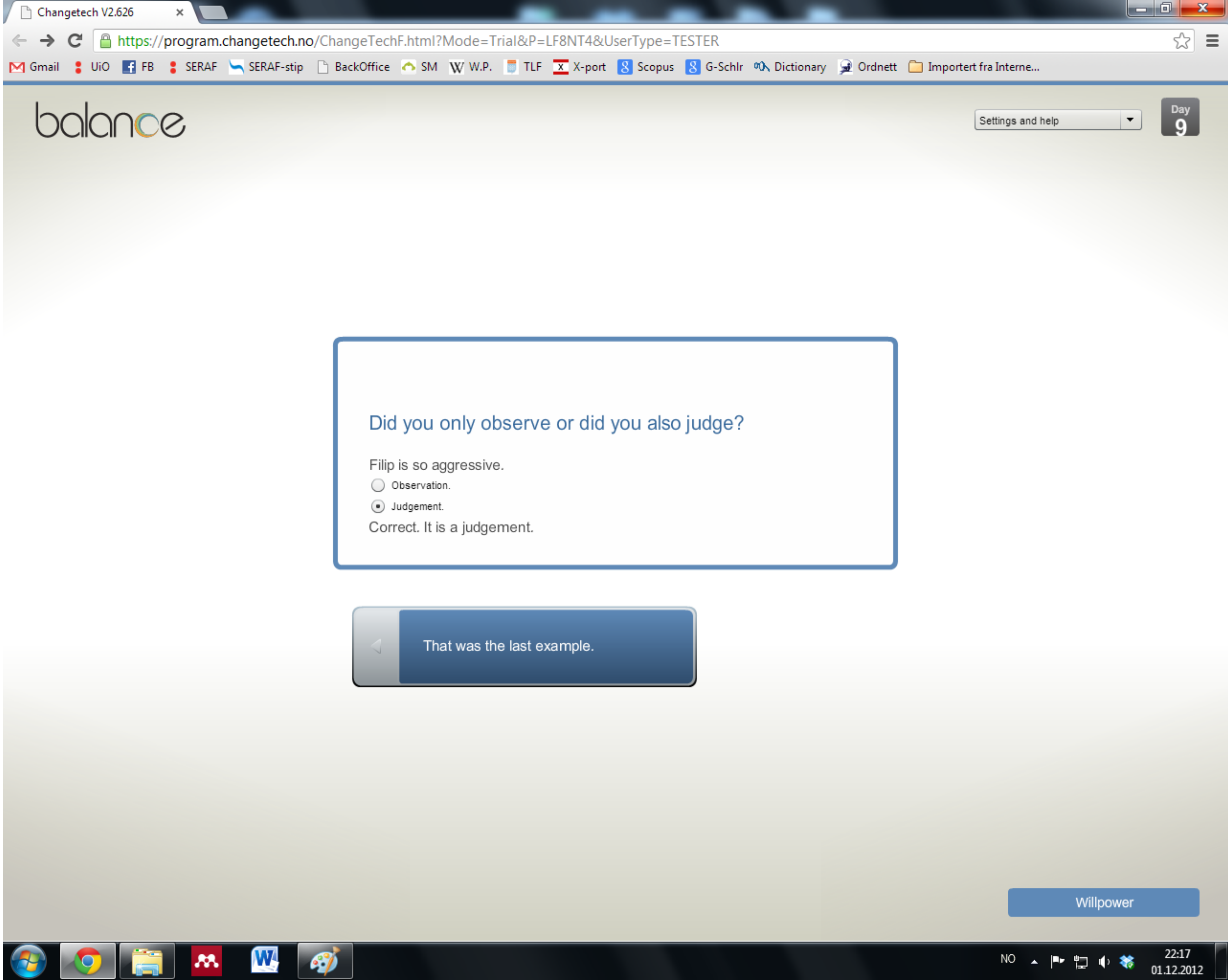
Changetech V2.626
https://program.changetech.no/ChangeTechF.html?Mode=Trial&P=LF8NT4&UserType=TESTER
Gmail UiO FB SERAF SERAF-stip BackOffice SM W.P. TLF X-port Scopus G-Schlr Dictionary Ordnett Importert fra Interne...
balance
Settings and help
Day 9
Did you only observe or did you also judge?
Filip is so aggressive.
Observation.
Judgement.
Correct. It is a judgement.
That was the last example.
Willpower
NO
22:17
01.12.2012

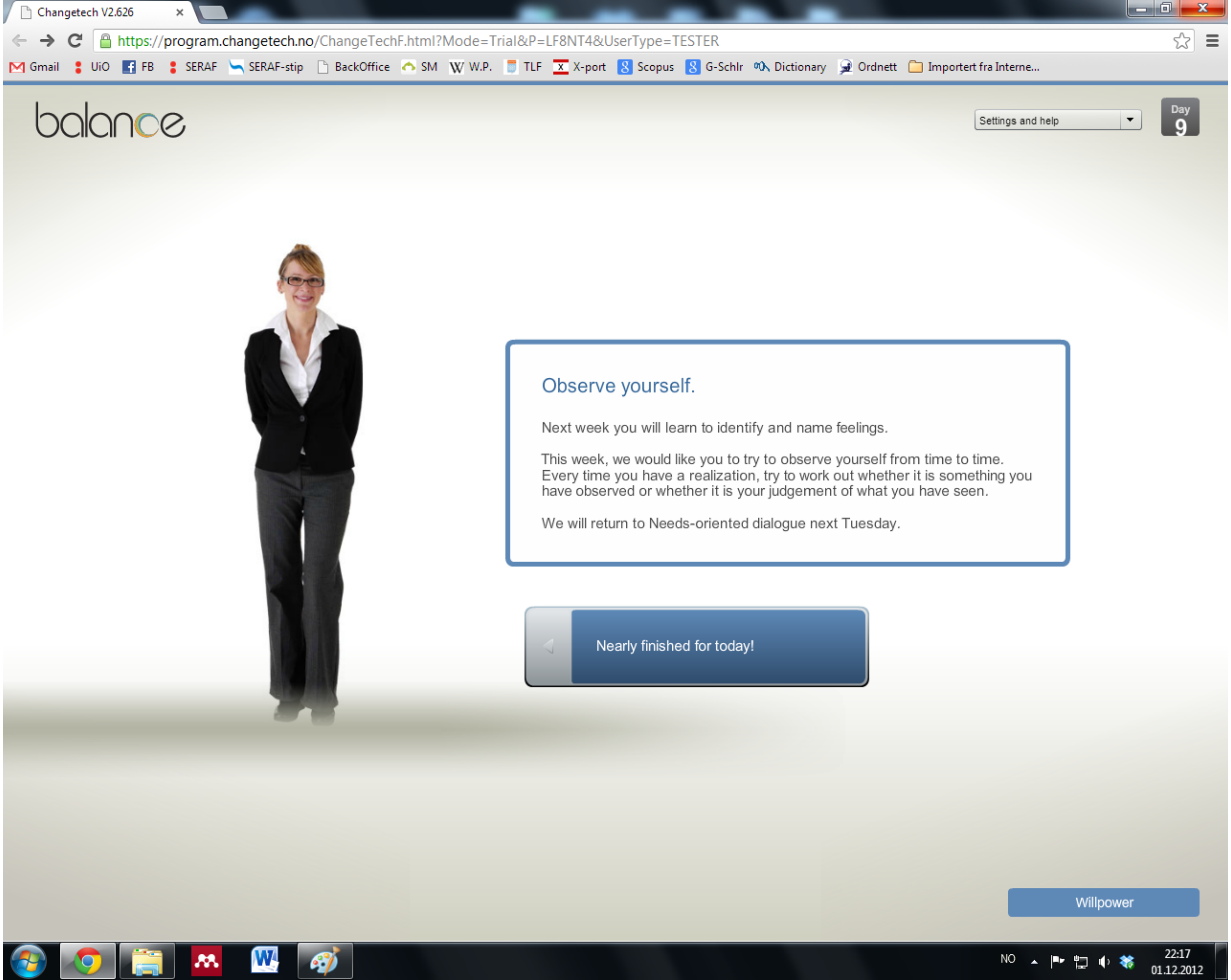
Changetech V2.626
https://program.changetech.no/ChangeTechF.html?Mode=Trial&P=LF8NT4&UserType=TESTER
Gmail UiO FB SERAF SERAF-stip BackOffice SM W.P. TLF X-port Scopus G-Schlr Dictionary Ordnett Importert fra Interne...
balance
Settings and help
Day 9
Observe yourself.
Next week you will learn to identify and name feelings.
This week, we would like you to try to observe yourself from time to time. Every time you have a realization, try to work out whether it is something you have observed or whether it is your judgement of what you have seen.
We will return to Needs-oriented dialogue next Tuesday.
Nearly finished for today!
Willpower
NO
22:17
01.12.2012

Positive psychology

Changetech V2.626

https://program.changetech.no/ChangeTechF.html?Mode=Trial&P=LF8NT4&UserType=TESTER

Gmail UiO FB SERAF SERAF-stip BackOffice SM W.P. TLF X-port Scopus G-Schlr Dictionary Ordnett Importert fra Interne...

balance

Settings and help

Day 4

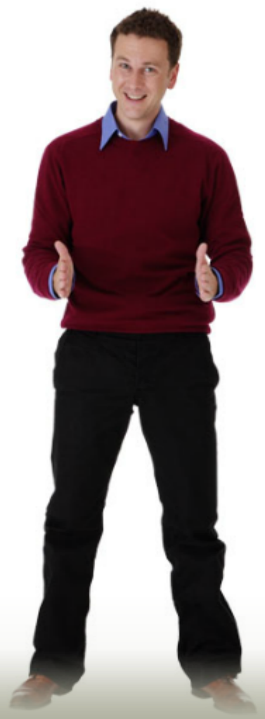

Every Wednesday we will give you a dose of positive psychology.

We started yesterday with a brief "mood report". Positive psychology is a new current in psychology that aims to give people who are basically healthy a stable, good mood and better quality of life.

The form of positive psychology that we use in Balance has been carefully tested in a range of studies.

One of the most common reasons for reverting to old habits is a sudden change in mood.

You might be thinking that positive psychology sounds a bit dubious, perhaps even scary, but it isn't. As you will soon discover.

You choose what you think suits you best

Mood

NO 21:37 01.12.2012

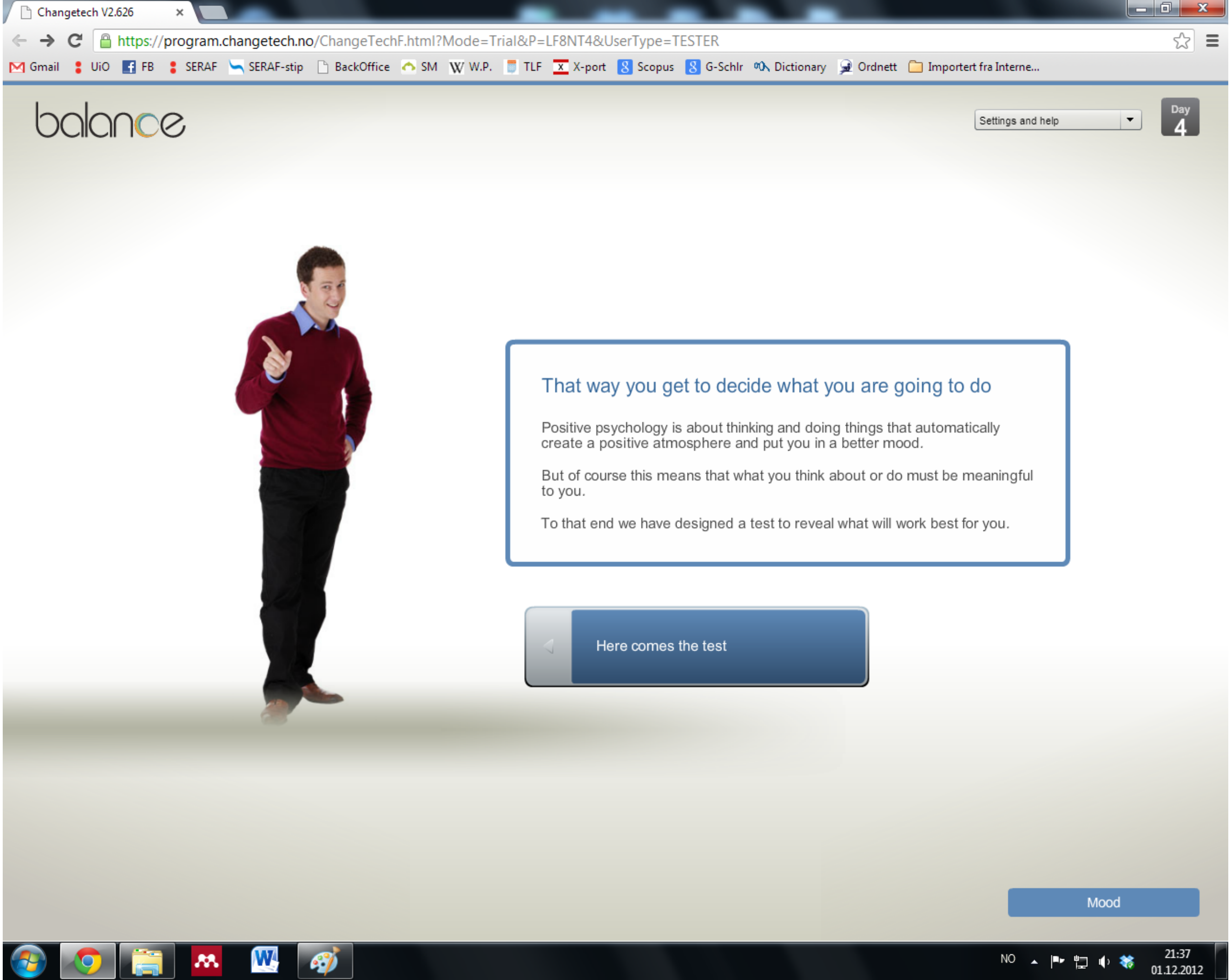
Changetech V2.626
https://program.changetech.no/ChangeTechF.html?Mode=Trial&P=LF8NT4&UserType=TESTER
Gmail UiO FB SERAF SERAF-stip BackOffice SM W.P. TLF X-port Scopus G-Schlr Dictionary Ordnett Importert fra Interne...
balance
Settings and help
Day 4
That way you get to decide what you are going to do
Positive psychology is about thinking and doing things that automatically create a positive atmosphere and put you in a better mood.
But of course this means that what you think about or do must be meaningful to you.
To that end we have designed a test to reveal what will work best for you.
Here comes the test
Mood
NO
21:37
01.12.2012

Changetech V2.626

https://program.changetech.no/ChangeTechF.html?Mode=Trial&P=LF8NT4&UserType=TESTER

Gmail UiO FB SERAF SERAF-stip BackOffice SM W.P. TLF X-port Scopus G-Schlr Dictionary Ordnett Importert fra Interne...

balance

Settings and help

Day 4

Thank you for your answers!

Based on your test results, it looks as though learning to challenge negative thoughts and turning them into more optimistic thoughts is meaningful to you and puts you in a good mood.

But perhaps you do not agree. The choice is yours.

The most important thing is that your choice is based on what interests you and will give you pleasure; do not choose what you think you "ought" to choose!

Choose here

Mood

NO 21:38 01.12.2012

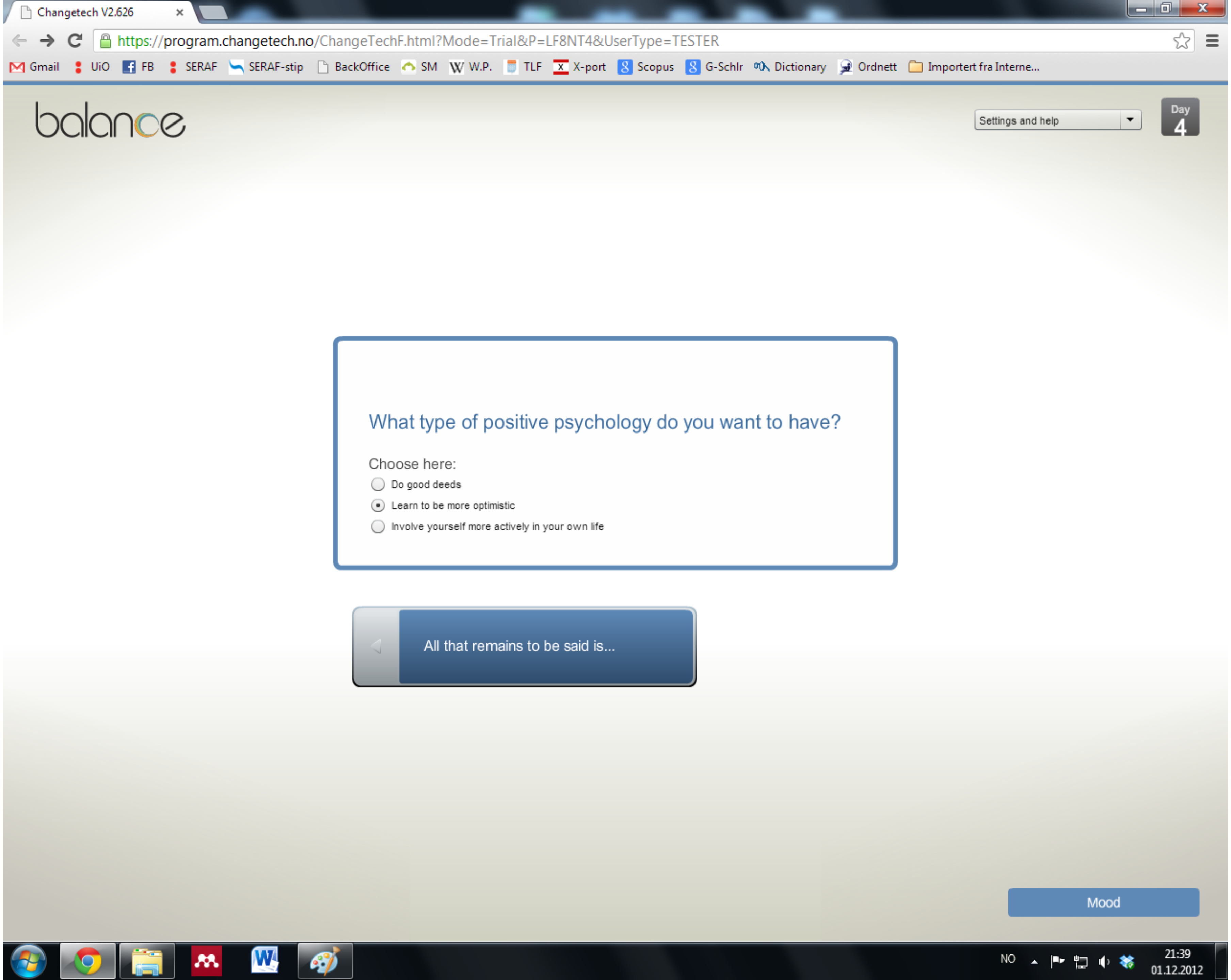
Changetech V2.626
https://program.changetech.no/ChangeTechF.html?Mode=Trial&P=LF8NT4&UserType=TESTER
Gmail UiO FB SERAF SERAF-stip BackOffice SM W.P. TLF X-port Scopus G-Schlr Dictionary Ordnett Importert fra Interne...
balance
Settings and help
Day 4
What type of positive psychology do you want to have?
Choose here:
Do good deeds
Learn to be more optimistic
Involve yourself more actively in your own life
All that remains to be said is...
Mood
NO
21:39
01.12.2012
