# Supplementary material for: Constructing a Theory- and Evidence-Based Treatment Rationale for Complex eHealth Interventions: Development of an Online Alcohol Intervention Using an Intervention Mapping Approach
Source: JMIR Res Protoc. 2013 Jan 23;2(1):e6. doi: 10.2196/resprot.2371 (PMC3629462; doi:10.2196/resprot.2371)
Supplement: Supplementary file 2 [file resprot_v2i1e6_app2.pdf]

Multimedia Appendix 2 – sample items from the weekly quiz

At the end of each week during the initial two months of Balance the clients are given a quiz that repeats the most important learning points from that week. As the clients tick one of the alternatives, they are given immediate feedback. If correct they will be affirmed and if they err they will receive an explanation and an encouragement to try once more. The clients are presented with one question per screen, and may navigate using the back or continue buttons. Below are some select items from various weeks.

The sample items are presented in this general format:

Question

- Alternative one
  - o Response from program if alternative one is ticked
- Alternative two
  - o Response from program if alternative one is ticked
- Alternative three
  - o Response from program if alternative one is ticked

Who are your main "allies" when you want to change habits?

- Willpower and motivation
  - o You will go a long way on willpower and motivation alone, but it will be hard work! Psst… being in a good mood makes things a lot easier for most people. Try again.
- Willpower and mood
  - o Willpower and mood are a strong team, but if you do not have the motivation to change, you will struggle. You will find yourself wondering "what's the point of trying to change?" Try again.

- Willpower, mood and motivation
  - That's right … if you build up your willpower, approach things with a smile and are motivated; there is little that can stop you achieving what you want! You have already started getting to know your "allies" during the past week.

What kind of people can develop drinking problems?

- Only people who are genetically disposed to alcoholism.
  - While it is true that genes do play a role, it is not only people who are genetically disposed to alcoholism who can develop drink problems. Choose another option.
- Only people who drink more than 4–6 glasses each time they drink.
  - You are right that if you drink 4–6 glasses of alcohol in a single session, you are increasing the risk of negative consequences (e.g. harm and accidents), but this does not mean that only people who drink 4–6 glasses can develop drinking problems.
- Everybody
  - Correct! Nobody can be inoculated against drinking problems.

Which of the following is essential when you are trying to change your drinking habits?

- Total abstinence.
  - Total abstinence can be a good goal for some people and may be helpful for a period before you adjust your consumption to the desired level, but there are other things that are more important ... Try again
- Being honest with yourself
  - Correct! If you are not honest with yourself, it is hard to keep track of your consumption and do something about the problem.

- Avoiding friends who drink and places which serve or sell alcohol
    - This can be a good strategy to help you achieve your goal for a while, but to be completely honest … you will find alcohol and people who drink everywhere, meaning is not a practical long-term strategy. Try again.

What do we mean by a slip-up?

- It is a relapse or regression
    - Almost, but not quite … A relapse or regression means that you have reverted to your old behaviour and, fortunately, this is not done in a single evening! Try again.
- A slip-up means you have failed
    - No, it doesn't. Unfortunately, we find it all too easy to think this way, but it is human to err and there is no need to make a mountain out of a molehill. Choose another option!
- A slip-up is just a slip-up, and I will carry on trying
    - You are completely right, of course! A spade is a spade and a slip-up is a slip-up – see it as an experience that you can learn from as you continue on your path towards your goal.

Can you name an important technique which can help you reach short-term and, hence, also your long-term goals?

- Knowledge about alcohol
    - Knowledge is a good thing. It can provide the motivation we need to want to change, but knowledge about alcohol alone will not help you attain your goals. Choose another option!
- Not thinking about the problem
    - This is perhaps the most popular technique that people use, although it

basically involves ignoring or avoiding the problem. The only problem with this technique is that the more you try not to think about the problem, the more you find yourself thinking about it. Try again!

- Pre-programming
  - Good! Merely focusing on your long-term goals can be rather disheartening, as success may seem a very long way off. Pre-programming is an effective planning technique which helps you keep moving towards your goals here and now and cope with problems that arise on the way.

What is stimulus control?

- Taking control and thinking about something else when faced with temptation.
  - Good thinking, but not entirely correct. Try again.
- Controlling the stimuli that make you want to drink, such as removing items that remind you of drinking.
  - Bull's eye! You cannot control absolutely everything, but it can be a great help to do everything you can to avoid being reminded or tempted to pour yourself a drink.
- Encouraging other people to drink, while keeping your own drinking under control.
  - Well, while we might all have done this on occasion, it is not what we call stimulus control. Try again.

What can you do to break a habit? (NB! There are several correct answers!)

- Change your surroundings
  - Correct! One aspect of habits is that they are done in familiar surroundings. This allows actions to be performed automatically without us thinking about what we are doing. If the surroundings are unfamiliar or new to you, your senses are sharpened and you think about what you are doing – and hey

presto, you are in control of the situation and can break free of the habit.

- Interrupt the sequence of choices which lead to an action
  - Correct! All habits consist of a series of decisions and actions that we perform. By stopping at one or more of these decisions or actions, we can stop ourselves from performing habitual actions. Have you ever gone into the kitchen to fetch a glass of milk and then the phone rings and after you have finished the call, you find yourself standing in the middle of the kitchen wondering why you are there and what you were actually doing?
- Make things more difficult
  - Correct! Habits are behaviour that we have repeated so many times it now seems natural and easy to us. If you want to break a habit, it is a good idea to challenge yourself and step outside your comfort zone. That way you force yourself to think about what you are doing and make a decision to break the habit.

What is impulse surfing?

- A surfing technique which involves acting on impulse.
  - A surfing technique which involves acting on impulse? What? An interesting, but bizarre concept! Try again!
- A technique which involves seeing impulsive urges as a wave and "surfing" them until they pass
  - Correct! Impulse surfing is a technique that helps you to notice cravings when they arise and deal with them. Cravings are short-term, and although they seem to come out of nowhere, there is nothing to say that you have to act on them. Accept that they are there and recognize them ... they will disappear all by themselves.
- A way of identifying impulsive behaviour.
  - Almost, but not quite. It does involve identifying impulsive urges and

cravings, but it is more than just that. Impulse surfing also helps you to resist the urges. Choose again!

Which of the following will make you most liked by other people?

- Taking the lead in conversation and sharing about myself
    - It is important to open up to others and share a bit about yourself, but there is a time and a place for everything. Generally, people who talk a lot about themselves tend to be less liked and have poorer relations with those around them. Choose another option.
- Letting the other person take the initiative and show interest in you
    - If the other person is also a good listener and understands you, you have a strong starting point for a good relationship, but you have to contribute too! Letting others take the initiative makes you more dependent on other people than is necessary to build up good relationships.
- Being a good listener and seeing the other person's point of view
    - Smart choice! Showing interest in other people, listening to what they have to say, asking questions and trying to see things from their point of view is a tried and tested recipe for good relations.

If you have had a drink or two more than you intended ...

- ... you might as well give up.
    - If, after a slip-up, you carry on sticking to what you have decided – what will that extra glass or two matter in the long run? A slip-up is just a slip-up!
- ... simply continue as before and you will succeed in changing your habits.
    - That's right, in the long run the occasional slip-up does not make much difference, provided you continue and stick to your goal!
- … it means you must stop drinking altogether and become a teetotaller.

    - It may be that you need to revise your goal slightly, but will setting such a strict goal really help? Think about it and try again!

Why is mood so important when you want to change your habits?

- Because you develop good habits when you are happy.
    - No, sadly. If only it were this simple! Try again!
- It is not important; this is a trick question.
    - No, it's not a trick question!
- Because you are more likely to slip up when you are feeling down and worn out.
    - Correct! When your mood is up, you are better able to withstand temptation and avoid slipping up.
