# Supplementary material for: Constructing a Theory- and Evidence-Based Treatment Rationale for Complex eHealth Interventions: Development of an Online Alcohol Intervention Using an Intervention Mapping Approach
Source: JMIR Res Protoc. 2013 Jan 23;2(1):e6. doi: 10.2196/resprot.2371 (PMC3629462; doi:10.2196/resprot.2371)
Supplement: Supplementary file 3 [file resprot_v2i1e6_app3.pdf]

Multimedia Appendix 3 - Just-in-time therapy for managing a recent lapse

Below is a transcript of a recorded conversation between a psychologist (P) and a client (C). The recording is provided to clients who report drinking above target the previous day.

P: Hello again... have a seat.

C: Thank you...

P: So... how are you doing?

C: (disappointed): (Sigh) badly...I've started drinking more.

P: (surprised): I see...(pause) What do you mean by that?

C: Well...I am drinking more than I should...for instance yesterday.

P: Ok. So you drank more than you had planned to yesterday. How do you feel now?

C: (Huh) I feel...well, I don't feel that great. I feel disappointed.

P: What do you mean by that?

C: Well...I am disappointed in myself, of course.

P: Mm...you feel disappointed in yourself. Why?

C: (Huh) It proves that I don't have any willpower and that I will never manage to change.

P: Ok. (Pause). So you drank more than you had planned. What happened?

C: Well... I went out with some friends in the evening, and had a great time.

P: Ok... So that's when you had a few drinks too many! What had your day been like? Had you not been thinking about alcohol at all?

C: He, he, well...actually, I had been wanting to go wild for a few days.

P: Ok, but what made you not do it before?

C: I had decided to change, and I managed to resist the temptation and stick to my plan.

P: Have you had difficult periods previously too?

C: Yes, of course. I have been tempted a number of times. I feel especially tempted

when I am exhausted or a bit frustrated.

P: So it's been hard a number of times. Yet you have managed to stick to your plan?

C: Well, yes, I have...

P: So you've actually proved to yourself that you have lots of willpower. Haven't you?

C: Well...yes...if you look at it that way, but apparently I didn't have any yesterday.

P: No, you drank more than you should have yesterday. But, from what you're telling me, yesterday you were in a situation in which most people would find it difficult not to have a relapse. Isn't that true?

C: Yes. That is probably true.

P: So you're actually very hard on yourself when you say you don't have any willpower.

C: Well...yes...I probably am.

P: Do you know what this tells me? It tells me that you do have lots of willpower and you do manage to stick to your plan. It was the situation you were in yesterday that caused your relapse. It became too demanding for you, and you had a momentary relapse. But why should this mean that you have started drinking too much again?

C: I don't know...maybe it was just a relapse. It probably was.

P: Right? The overall situation you were in caused the relapse. That doesn't at all prove that you don't have any willpower. After all, your willpower had been sufficiently strong for days.

C: Yes, if you look at it that way...

P: That's how it is for everyone who has a relapse. That's why it's called a relapse A specific situation causes it. You're exhausted and a bit depressed, or you've had an uncomfortable experience. Perhaps there is something about the situation that is tempting.

But this will pass. Is there any reason you shouldn't just continue with your plan, given that the specific situation yesterday has passed?

C: (Huh) No, I guess not.

P: So what does that mean?

C: Well...(laughs) it means that you win, doesn't it?

P: (Laughs a bit) You win, don't you? To my mind, you're actually stronger than ever. You have shown that you can continue with your plan after a relapse, and you have learnt from this experience. So who's the winner?

C: If I manage, in the end I'll be the winner.

P: So what do you think about that...are you more or less likely to succeed now?

C: Well, when you look at it that way, I am actually more likely to succeed.
